# SHP2 regulates VEGFR2 Y1175/PLCγ signaling to impair tumor endothelial barrier stability

**DOI:** 10.1016/j.isci.2026.114784

**Published:** 2026-01-27

**Authors:** Polina Kremmyda, Sara Owad, Sagnik Pal, Elvira Wildheim, Catarina Chanoca, Cecilia Lindskog, Elin Sjöberg

**Affiliations:** 1 Department of Immunology, Genetics and Pathology, Beijer and Science for Life Laboratories, Uppsala University, Uppsala, Sweden

**Keywords:** microenvironment, molecular biology, cell biology, cancer

## Abstract

Tumor vasculature is abnormally formed, with an endothelial cell (EC) barrier lacking integrity, resulting in hyperpermeable vessels. Elevated VEGFA levels drive the formation of the dysfunctional vasculature by activating VEGFR2 signaling. The VEGFR2 tyrosine site pY1175 was recently shown to stimulate a PLCγ/eNOS/Src pathway, promoting vascular leakage and hindering therapeutic targeting and anti-tumor immunity. High endothelial PLCγ levels correlated to poor kidney cancer prognosis, which indicated endothelial PLCγ as a prognostic biomarker. In this study, we reveal SHP2 as a binding partner of pY1175 and show that SHP2 cooperates with PLCγ to mediate VEGFA-induced permeability in both healthy and tumor vasculatures. Targeting the VEGFR2/PLCγ/SHP2 axis—genetically or pharmacologically—reduces EC junctional phosphorylation to prevent VE-cadherin internalization, followed by reduced macromolecular leakage. Tumor EC expression of PLCγ or SHP2 is associated with vascular leakage in human kidney cancer, underscoring their potential as targets for vascular normalization and biomarkers for disease progression and treatment response.

## Highlights

• The phosphatase SHP2 forms a complex with PLCγ at VEGFR2 pY1175 in endothelial cells
• VEGFR2/PLCγ/SHP2 signaling activates eNOS/Src, causing VEC disruption and leakage
• Targeting the pathway stabilizes the barrier in both healthy and tumor vasculatures
• PLCγ or SHP2 expression in human RCC vessels correlates with enhanced leakage

## Graphical abstract

**Figure:**
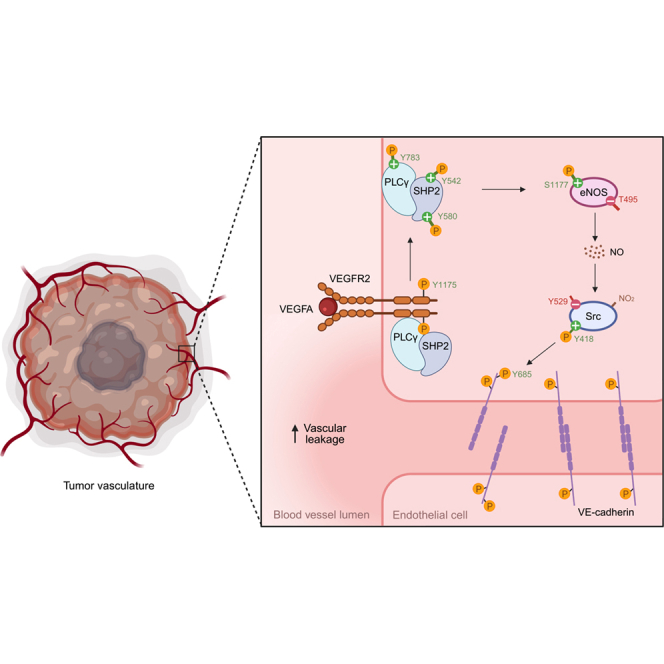

## Introduction

Solid tumors are dependent on the ability to form new blood vessels for their growth and progression. Sustained tumor angiogenesis, one of the hallmarks of cancer,^1^ is driven by increased hypoxia. As a consequence, the abnormally formed blood vessels possess a defective endothelial cell (EC) barrier with dysregulated endothelial cell junctions and macromolecular leakage, resulting in increased edema and interstitial fluid pressure.^2^ Loss of barrier integrity further alters immune and tumor cell trafficking across the vessel wall. This prevents drug delivery, attenuates anti-tumor immunity, and promotes metastasis.^2^^,^^3^ Increased hypoxia in the tumor microenvironment leads to chronic production of the leakage agonist vascular endothelial growth factor A (VEGFA), which signals via its main receptor VEGFR2 to promote growth of the hyperpermeable tumor vasculature.^2^^,^^3^^,^^4^

By pharmacological targeting of VEGFA or VEGFR2 in cancers, the vasculature can be normalized and the EC barrier stabilized, resulting in lower interstitial fluid pressure and enhanced vessel perfusion, accompanied by decreased metastatic spread and enhanced infiltration and activation of immune cells.^5^^,^^6^ Targeting VEGFR2 has shown clinical relevance for certain tumors, including clear cell renal cell carcinoma (ccRCC), the most prevalent form of kidney cancer.^7^ ccRCC is one of the most immunogenic^8^ and vascularized cancers, characterized by aberrant hypoxia regulation and enhanced levels of VEGFA.^9^^,^^10^ While vascular normalization therapies have shown promise, not all patients respond to therapy, and complete inhibition of VEGFR2 signaling can also have the reverse effect, making the tumor vasculature even more abnormal, leading to drug resistance.^11^ Instead of blocking VEGFR2 signaling, targeting individual downstream signal transduction pathways and their corresponding endothelial cell behaviors is an attractive therapeutic approach. It is also of relevance to identify which patients would benefit from currently available cancer therapies.

The healthy EC barrier is kept intact via cell-cell junctions including adherens junctions, of which vascular endothelial-cadherin (VEC) is the main component, that regulates barrier integrity of peripheral blood vessels.^12^ In response to inflammatory cytokines, including histamine, bradykinin, and thrombin or VEGFA, the barrier becomes compromised.^13^ Endothelial cells respond to these stimuli via phosphorylation of VEC at specific tyrosine (Y) residues, leading to internalization and ubiquitination, with vascular leakage as a consequence.^13^^,^^14^^,^^15^ In addition, studies have shown individual functions of the VEC tyrosine residues Y658, Y685, and Y731, with specific regulatory effects on leakage and immune cell transmigration.^15^^,^^16^^,^^17^ However, studies in mice with VEC point mutations have shown that tyrosine phosphorylation alone is not sufficient to regulate the barrier.^15^^,^^16^^,^^18^ The full composition and regulation of adherens junctions is still not known, and there are unidentified proteins and pathways that affect barrier integrity.

VEGFA binding to VEGFR2 causes phosphorylation of several tyrosine residues, including Y951 in the kinase insert, Y1054/Y1059 in the kinase domain, and Y1175 and Y1214 at the C-terminus (human sequences). Studies in mouse models with inactivating mutations of these phosphosites have demonstrated activation of distinct downstream signaling pathways and endothelial cell functions.^19^^,^^20^^,^^21^ The Y949 and Y1173 (human Y951 and Y1175) phosphosites mediate activation of Src family kinases (SFKs), including Src, leading to vascular permeability and metastatic spread in experimental tumor models.^19^^,^^21^^,^^22^ In addition, signaling downstream of the Y1175 phosphosite (pY1175) is necessary for endothelial progenitor differentiation through activation of phospholipase Cγ (PLCγ).^23^^,^^24^ Upon activation of PLCγ, diacylglycerol (DAG) stimulates protein kinase C (PKC), initiating extracellular regulated kinases 1/2 (ERK1/2) signaling and promoting endothelial proliferation.^25^ Concurrently, inositol 1,4,5 trisphosphate production results in calcium release from the endoplasmic reticulum (ER), contributing to vascular permeability.^19^ We have recently identified a clinically relevant fundamental role of pY1175/PLCγ signaling in activating endothelial nitric oxide synthase (eNOS)/Src signaling to regulate the EC barrier of tumor vessels. Upon eNOS-induced Src activation, tyrosine phosphorylation of VEC at Y685 was enhanced, accompanied by disintegration of EC barrier adherens junctions. This was followed by enhanced vessel leakage and infiltration of B cells, T cells, immunosuppressive regulatory T cells (Tregs), and cytokines.^19^ Additionally, PLCγ was identified as a biomarker for an abnormal vasculature and poor patient outcome in kidney cancer.^19^

Here, we further expand upon earlier findings of the clinical relevance and importance of vascular PLCγ in tumor vascular leakage. Mechanistically, an interaction partner for pY1175/PLCγ is identified—SHP2, which exhibits both phosphatase-dependent and -independent functions in its interplay with PLCγ. The pathway operates in both healthy and tumor endothelium, which is of relevance for macromolecular leakage, anti-tumor immunity, and patient survival.

## Results

### SHP2 is identified as a novel PLCγ-dependent VEGFR2 pY1175 interaction partner in endothelial cells

**Figure 1:**
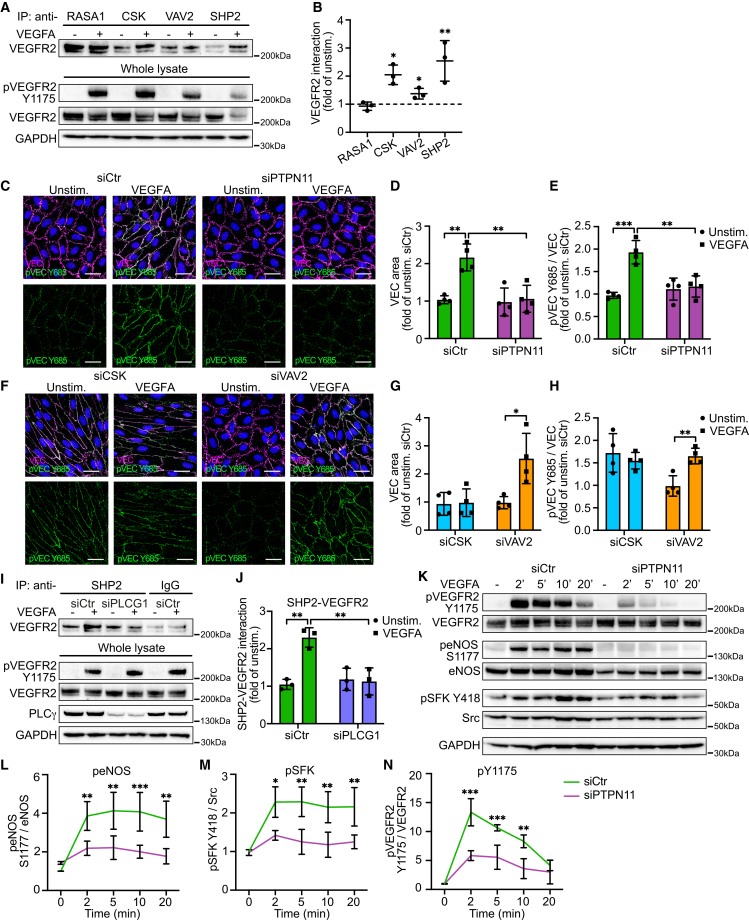
SHP2 is identified as a PLCγ-dependent VEGFR2 pY1175 interaction partner in endothelial cells (A) Representative western blots showing VEGFR2 immunoprecipitated with antibodies against RASA1, CSK, VAV2, or SHP2 in HUVECs unstimulated (−) or stimulated (+) with VEGFA (100 ng/mL, 5 min). Corresponding whole-cell lysates analyzed using antibodies against phosphorylated VEGFR2 (pY1175), total VEGFR2, and GAPDH as loading control. (B) Quantification of VEGFR2 interaction with RASA1, CSK, VAV2, and SHP2 from (A); *n* = 3 independent experiments. (C) Representative images of immunostainings for VEC (magenta), pVEC Y685 (green), and DAPI (blue) of unstimulated or VEGFA-stimulated (100 ng/mL, 5 min) HUVECs, pretreated with *siCtr* or *siPTPN11.* Scale bars: 30 μm. (D and E) Quantifications of MFI from (C), displayed as fold change relative to unstimulated control. (D) MFI of the VEC area. (E) MFI of pVEC Y685; *n* = 3 independent experiments, ≥3 fields of view per experiment. (F) Representative images of immunostainings for VEC (magenta), pVEC Y685 (green), and DAPI (blue) of unstimulated or VEGFA-stimulated (100 ng/mL, 5 min) HUVECs, pretreated with *siCSK* or *siVAV2.* Scale bars: 30 μm. (G and H) Quantifications of MFI from (F), shown as fold change over unstimulated control; *n* = 3 independent experiments, ≥3 fields of view per experiment. (I) Representative western blot showing VEGFR2 immunoprecipitated with antibodies against SHP2 or IgG control from unstimulated or VEGFA-stimulated (100 ng/mL, 5 min) HUVECs, pre-treated with *siCtr* or *siPLCG1*. Corresponding whole-cell lysates analyzed by blotting with antibodies against pVEGFR2 Y1175, VEGFR2, PLCγ, and GAPDH as loading control. (J) Quantification of VEGFR2-SHP2 binding from (I); *n* = 3 independent experiments. (K) Representative western blot showing downstream VEGFA-induced signaling in unstimulated (−) or 100 ng/mL VEGFA-stimulated HUVECs, for 2, 5, 10, and 20 min pretreated with siCtr or siPTPN11. (L–N) Quantification of western blots from (K), shown as fold change relative to unstimulated control. (L) Quantification of western blots for peNOS S1177. (M) Quantification of western blots for pSFK Y418. (N) Quantification of western blots for pVEGFR2 Y1175; *n* = 4–5 independent experiments. One-way ANOVA. Data represent the mean ± SD. ∗, *p* < 0.05; ∗∗, *p* < 0.01; ∗∗∗, *p* < 0.001. HUVECs, human umbilical vein endothelial cells; VEC, VE-cadherin; MFI, mean fluorescence intensity. See also Figure S1.

The interactome of the pY1175 phosphosite was recently analyzed by an unbiased mass spectrometry (MS) approach, and PLCγ was discovered as an interaction partner, crucial for VEGFA-induced vascular permeability.^19^ In the current study, we aimed to further explore the mechanistic impact of how VEGFR2 Y1175 signaling stimulates vascular permeability. In addition to PLCγ, the MS results revealed potential interaction partners of the phosphosite,^19^ including RAS P21 protein activator 1 (RASA1),^26^ Src homology region 2 domain-containing phosphatase 2 (SHP2; gene name *PTPN11*),^16^^,^^17^ the guanine nucleotide exchange factor VAV2,^18^ and the C-terminal Src kinase CSK.^27^ Little is known about these interaction candidates’ involvement in VEGFR2 signaling and the downstream endothelial cell responses. This urged us to explore whether they exhibit specific functions in VEGFA-stimulated vascular permeability. To validate the MS results, a co-immunoprecipitation (coIP) was performed between candidates and total VEGFR2. Upon VEGFA stimulation, an increased interaction was seen between VEGFR2 and SHP2, CSK, or VAV2 (Figures 1A and 1B). Prominent binding between VEGFR2 and RASA1 was also identified, but no increased interaction upon receptor activation was found (Figures 1A and 1B), and therefore, RASA1 was excluded from additional analyses.

Under resting conditions, VEC forms stable, linear junctions, which transform into a broader, jagged morphology upon stimulation with VEGFA or inflammatory mediators such as histamine and bradykinin. This is accompanied by increased phosphorylation of pVEC Y685 and disintegration of adherens junctions.^12^^,^^15^^,^^19^ To elucidate the involvement of each candidate in VEGFA-induced vascular permeability, human umbilical vein endothelial cells (HUVECs) with siRNA-mediated downregulation of each candidate (Figures S1A–S1D) were immunostained for VEC and pVEC Y685. Downregulation of SHP2 significantly suppressed pVEC Y685 signaling and VEC disruption in response to VEGFA (Figures 1C–1E), which was not seen by downregulation of CSK or VAV2 (Figures 1F–1H). Interestingly, CSK downregulation instead stabilized straight linear junction with enhanced basal levels of pVEC Y685. This is in accordance with the known role of CSK as a negative regulator of SFK members via phosphorylation of the inhibitory Y529 site,^27^^,^^28^ together with the function of SFK members to phosphorylate VEC residues *in vivo*.^19^^,^^29^ Notably, CSK has previously been shown to bind to the Y685 site of VEC^27^ and was recently demonstrated to control leukocyte extravasation without altering VEGFA- or histamine-stimulated vascular permeability *in vivo*.^28^ This unexpected finding is most likely linked to CSK’s role in inactivation of both Src and Yes, two SFKs with opposite functions in regulating EC barrier integrity.^29^

The findings of PLCγ and SHP2 binding to the VEGFR2 pY1175 phosphosite prompted us to explore their interplay. A coIP of VEGFR2 and SHP2 was, therefore, performed in HUVECs pre-treated with *siPLCG1* or *siCtr*. The results showed that the VEGFA-induced SHP2-VEGFR2 interaction was abolished upon *siPLCG1* treatment. Accordingly, SHP2 was recruited to VEGFR2 upon ligand binding, which only occurred in the presence of PLCγ (Figures 1I and 1J). Based on our results of both PLCγ and SHP2 interacting with VEGFR2, we hypothesized that SHP2, PLCγ, and VEGFR2 would form a complex in endothelial cells upon receptor activation. To test if PLCγ and SHP2 also engaged in a complex, a coIP was performed. The results showed the interaction of PLCγ and SHP2 (Figures S1E and S1F), further in concordance with what has previously been reported in the literature for additional cell types and RTKs.^30^^,^^31^ Notably, an increased ligand-dependent interaction was also seen for CSK and PLCγ (Figures S1E and S1F), a finding that would require further validation and investigation to reveal functional outcomes in endothelial cells. In addition, we were intrigued to investigate the cellular distribution of VEGFA-induced complex formation of PLCγ and SHP2 and performed a proximity ligation assay (PLA) for phosphorylated SHP2 and PLCγ. The results confirmed an enhanced interaction of pSHP2 (Y542) and PLCγ upon VEGFA stimulation, in particular at the plasma membrane (Figures S1G and S1H).

VEGFR2 Y1175/PLCγ-signaling has been shown to activate the eNOS/Src pathway, resulting in opening of the EC barrier to allow the passage of molecules and cells.^19^ To investigate whether downregulation of SHP2 affected VEGFR2 Y1175/PLCγ/eNOS/Src signaling, siRNA-mediated targeting was performed in HUVECs prior to VEGFA treatment over time. Western blot analysis showed that SHP2 downregulation abolished phosphorylation of the activating S1177 site of eNOS and the Y418 site of SFK (Figures 1K–1M), demonstrating the presence of SHP2 being critical for eNOS and SFK activation. The results also showed reduced phosphorylation of pVEGFR2 Y1175 (Figures 1K and 1N) and pPLCγ Y783 (Figures S1I and S1J) upon SHP2 knockdown, suggesting that SHP2 exerts functions both upstream and downstream of PLCγ. Activation of VEGFR2 also enhances MAPK and PI3K signaling, followed by altered endothelial cell behaviors.^3^ Although pERK or pAKT levels and kinetics were affected, there was no significant difference upon SHP2 downregulation (Figures S1I, S1K, and S1L).

In conclusion, SHP2 is identified as a pY1175 interaction partner that binds to VEGFR2 in a PLCγ-dependent manner, forming a complex with PLCγ and activating downstream eNOS and Src signaling, which are crucial for vascular leakage.

### Endothelial PLCγ/SHP2-signaling mediates activation of Src by regulation of both the activating and inhibitory tyrosine phosphorylation sites

**Figure 2:**
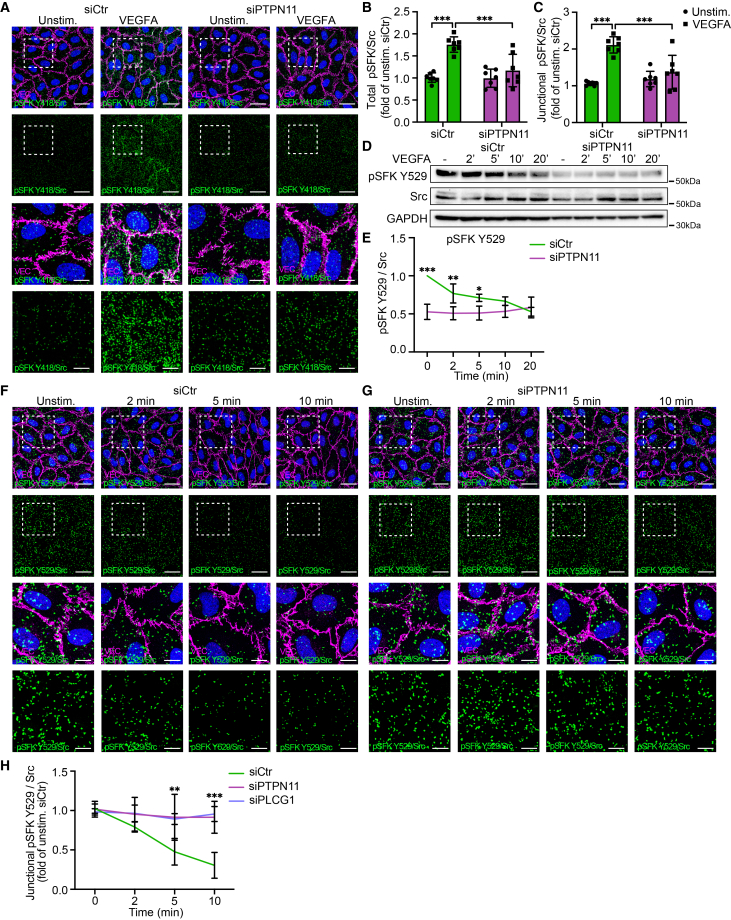
Endothelial PLCγ/SHP2 signaling mediates activation of Src by regulation of both the activating and inhibitory tyrosine phosphorylation sites (A) PLA using antibodies against Src and pSFK Y418 to detect phosphorylation of Src on Y418 in unstimulated or VEGFA-stimulated HUVECs (100 ng/mL, 5min), pretreated with *siCtr* or *siPTPN11*. Endothelial junctions are stained for VEC (magenta) and nuclei with DAPI (blue). Scale bars: 30 μm. Boxed regions in the upper panels are shown at higher magnification in panels below. Scale bars: 10 μm. (B and C) MFI quantifications from (A), displayed as fold change relative to unstimulated control. (B) MFI of the total PLA signal. (C) MFI of the junctional PLA signals representing Y418 phosphorylation of Src; *n* = 7 independent experiments, ≥3 fields of view per experiment. (D) Representative western blot showing pSFK Y529 signaling in unstimulated (−) or VEGFA-stimulated HUVECs (100 ng/mL) for 2, 5, 10 and 20 min, pretreated with *siCtr* or si*PTPN11*. (E) Quantification of western blots from (D); *n* = 5 independent experiments. (F and G) PLA using antibodies against Src and pSFK 529, visualizing phosphorylation of Src at the inhibitory phosphosite in HUVECs stimulated for 2, 5, and 10 min or left unstimulated. (F) Phosphorylation of Src at the inhibitory phosphosite in HUVECs pre-treated with *siCtr*. (G) Phosphorylation of Src at the inhibitory phosphosite in HUVECs pre-treated with *siPTPN11*. Endothelial junctions are stained for VEC (magenta) and nuclei with DAPI (blue). Scale bars: 30 μm. Boxed regions in the upper panels are shown at a higher magnification in panels below. Scale bars: 10 μm. (H) Quantification of PLA experiments from (F) and (G) and Figure S2F; *n* = 6 independent experiments, ≥3 fields of view per experiment. One-way ANOVA. Data represent the mean ± SD. ∗, *p* < 0.05; ∗∗, *p* < 0.01; ∗∗∗, *p* < 0.001. PLA, proximity ligation assay; HUVECs, human umbilical vein endothelial cells; VEC, VE-cadherin. See also Figure S2.

The pSFK Y418 antibody used for western blot in this study recognizes all SFK members due to their structural similarity. We, therefore, performed PLAs to specifically look at the activation of either Src or Yes, using antibodies against pSFK Y418 combined with either Src or Yes antibodies. PLA results showed enhanced VEGFA-stimulated activation of total and junctional Src, assessed by the quantification of PLA complexes, which was decreased upon siRNA-mediated downregulation of SHP2 (Figures 2A–2C). Instead, activation of Yes was unaffected by VEGFA treatment, as previously seen,^19^ and SHP2 knockdown had no effect (Figures S2A–S2C).

To further explore Src activation downstream of PLCγ/SHP2 signaling, phosphorylation of the inhibitory site Y529 was investigated using western blot. In *siCtr*-treated cells, VEGFA stimulation decreased the phosphorylation of SFK Y529 over time (Figures 2D and 2E). In cells treated with *siPTPN11* (Figures 2D and 2E) or *siPLCG1* (Figures S2D and S2E), the VEGFA-induced decrease in pSFK Y529 was absent. Unexpectedly, there was already a decrease at basal level, which might correspond to the antibody recognizing all SFK members. Therefore, a PLA with antibodies recognizing Src and pSFK Y529 was employed. The results confirmed the abolished VEGFA-induced dephosphorylation of pSrc Y529 upon downregulation of either SHP2 or PLCγ and did not show altered basal phosphorylation (Figures 2F–2H and S2F).

Conclusively, SHP2/PLCγ signaling enhances Src activation by increasing both phosphorylation of the Y418 activating site and dephosphorylation of the inhibitory Y529 site.

### PLCγ/SHP2 interplay leads to eNOS activation followed by Src nitration

**Figure 3:**
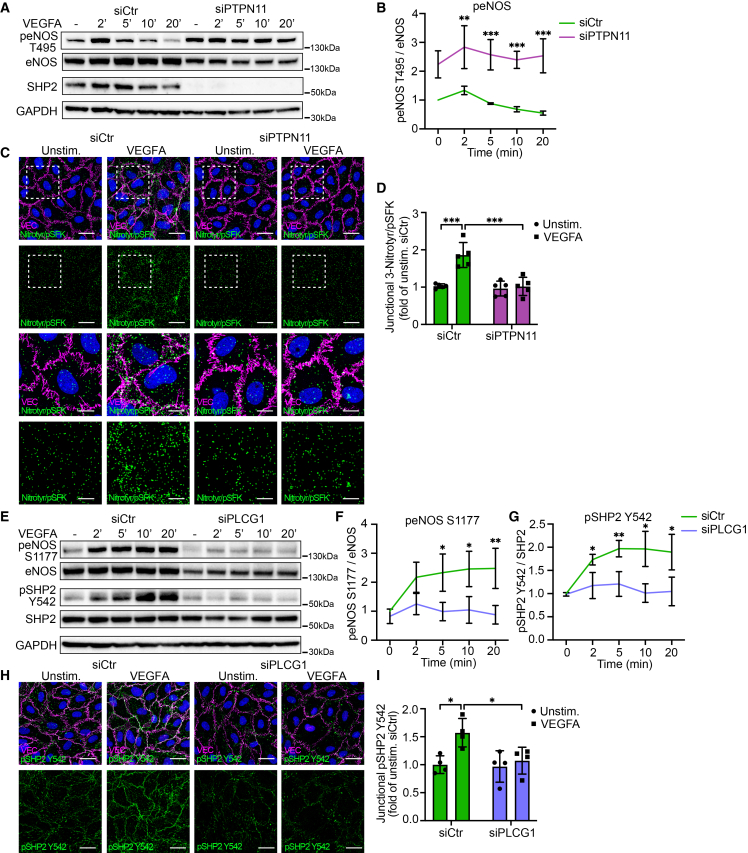
PLCγ/SHP2 interplay leads to eNOS activation followed by Src nitration (A) Representative western blot showing eNOS T495 signaling in unstimulated (−) or 100 ng/mL VEGFA-stimulated HUVECs for 2, 5, 10 and 20 min, pre-treated with *siCtr* or *siPTPN11*. (B) Quantification of western blots from (A); *n* = 4 independent experiments. (C) PLA for NitroTyr and pSFK Y418 to detect full activation of Src in HUVECs stimulated with VEGFA (100 ng/mL, 5 min) or left unstimulated, and pretreated with *siCtr* or *siPTPN11*. Endothelial junctions are stained for VEC (magenta) and DAPI (blue). Scale bars: 30 μm. Boxed regions in the upper panels are shown at higher magnification in panels below. Scale bars: 10 μm. (D) Quantification of junctional MFI PLA signals representing Y418 phosphorylation and 3-nitration of Src from (C), displayed as fold change to unstimulated control; *n* = 5 independent experiments, ≥3 fields of view per experiment. (E) Western blot showing eNOS S1177 and SHP2 Y542 signaling in unstimulated (−) or 100 ng/mL VEGFA-stimulated HUVECs for 2, 5, 10, and 20 min, pre-treated with *siCtr* or *siPLCG1*. (F and G) Quantifications of western blots from (E). (F) Quantifications of western blots for peNOS S1177. (G) Quantifications of western blots for pSHP2 Y542; *n* = 4 independent experiments. (H) Representative immunostaining images with antibodies against VE-cadherin (VEC; magenta) and pSHP2 Y542 (green), in HUVECs unstimulated or stimulated with VEGFA (100 ng/mL, 5min) after downregulation with *siCtr* or *siPLCG1*. Scale bars: 30 μm. (I) Quantification of MFI from (H), shown as fold change relative to unstimulated control; *n* = 4 independent experiments, ≥3 fields of view/experiment. One-way ANOVA. Data represent the mean ± SD. ∗, *p* < 0.05; ∗∗, *p* < 0.01; ∗∗∗, *p* < 0.001. VEC, VE-cadherin. See also Figure S3.

The prominent decrease in eNOS signaling subsequent to abolished PLCγ/SHP2 signaling prompted further mechanistic insights in eNOS activation and signaling. The phosphorylation of the eNOS inhibitory site T495 (peNOS T495) was, therefore, analyzed. The results demonstrated VEGFA-induced T495 dephosphorylation over time (with a minor increase after 2 min), which did not occur upon the downregulation of SHP2 (Figures 3A and 3B). Instead, in the absence of SHP2 signaling, there was a stable increase in peNOS T495, indicating loss of activation in response to VEGFA (Figures 3A and 3B). Similar findings were also observed in PLCγ-downregulated endothelial cells (Figures S3A and S3B).

We previously identified that VEGFA-regulated PLCγ signaling led to eNOS activation followed by enhanced Src nitration.^19^ Here, we further show that PLCγ/SHP2 plays an important role in nitration of Src. Nitration of both total (Figures S3C–S3E) and active Src (Figures 3C and 3D) in response to VEGFA was decreased in endothelial cells upon SHP2 downregulation. Together, these data support the downstream regulation of eNOS and Src activation by pY1175/PLCγ/SHP2 signaling. To further explore the interplay of PLCγ and SHP2, western blot and immunostainings for SHP2 and phosphorylated SHP2 were performed in HUVECs treated with siRNAs targeting scrambled control or PLCγ. Analysis of data confirmed that the downregulation of PLCγ (Figures S3F and S3G) reduced VEGFA-dependent eNOS activation^19^ (Figures 3E and 3F). In addition, phosphorylation of SHP2 at tyrosine Y542 (Figures 3E and 3G) and Y580 (Figures S3F and S3H), particularly at endothelial junctions (Figures 3H, 3I, S3I, and S3J), was also reduced upon PLCγ silencing. Subsequent to VEGFA stimulation, the cellular distribution of SHP2 was also altered, and localization of SHP2 to the plasma membrane was abolished upon PLCγ downregulation (Figures S3K and S3L).

Presence of PLCγ is, therefore, crucial for VEGFA-stimulated phosphorylation and recruitment of SHP2 to the plasma membrane, followed by eNOS/Src signaling, and we conclude that the interplay between the two proteins is needed for downstream signaling.

### VEGFR2 pY1173/PLCγ-induced vascular permeability *in vivo* requires SHP2

**Figure 4:**
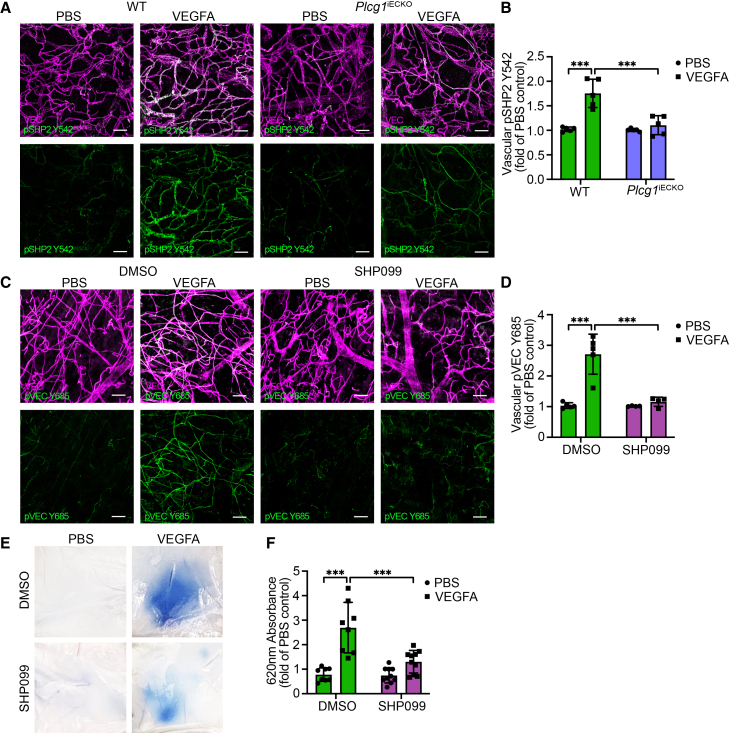
VEGFR2 pY1173/PLCγ-induced vascular permeability *in vivo* requires SHP2 (A) Representative immunostaining images with antibodies against VEC (magenta) and pSHP2 Y542 (green) in the back skin of WT and *Plcg1*^*iECKO*^ mice after intradermal injection of PBS or VEGFA. Scale bars: 50 μm. (B) Quantification of MFI values from (A), for vascular pSHP2 Y542, displayed as fold change relative to PBS control; *n* = 5 mice/genotype, ≥3 fields of view/mouse. (C) Representative images of immunostaining for VEC (magenta) and pVEC Y685 (green) in the back skin of WT mice, intradermally injected with DMSO or SHP099 and subsequently PBS or VEGFA at the same site. Scale bars: 50 μm. (D) Quantification of MFI values from (C), for vascular pVEC Y685, shown as fold change relative to PBS control; *n* = 5 (DMSO) and 4 (SHP099) WT mice, ≥3 fields of view/mouse. (E) The Miles assay showing Evans blue leakage in the back skin of DMSO (control) or SHP099 treated WT mice, intradermally injected with PBS or VEGFA. (F) Quantification of extravasated Evans blue from (E), shown as fold change of DMSO-PBS-treated mice; *n* ≥ 8 mice/condition. One-way ANOVA. Data represent the mean ± SD. ∗, *p* < 0.05; ∗∗, *p* < 0.01; ∗∗∗, *p* < 0.001. VEC, VE-cadherin. See also Figure S4.

To examine the interplay between PLCγ and SHP2 downstream of VEGFR2 pY1173 *in vivo* in the healthy endothelium, *Plcg1*^*iECKO*^ and wild-type (WT) mice were injected intradermally with VEGFA or PBS in the dorsal back skin. Subsequent to vessel perfusion, the skin was excised, and immunostaining for pSHP2 Y542 was performed. VEGFA treatment resulted in a significant increase in the vascular pSHP2 Y542 levels in WT mice as compared to PBS treatment, not seen in *Plcg1*^*iECKO*^ mice (Figures 4A and 4B). Thus, SHP2 phosphorylation in response to VEGFA requires active PLCγ signaling in endothelial cells both *in vitro* and *in vivo*.

Small molecule pharmacological inhibitors targeting SHP2 have been developed and validated.^32^ Here, we used the allosteric inhibitor SHP099, which stabilizes SHP2 in an autoinhibitory enzymatically inactive state,^32^^,^^33^ to assess how the acute inhibition of SHP2 affects endothelial cell signaling and vascular leakage. Western blot analysis demonstrated that SHP099 treatment of HUVECs resulted in decreased peNOS S1177 and pSFK Y418 levels in response to VEGFA stimulation compared with DMSO treatment (Figures S4A–S4C). In addition, VEGFA-induced release of the inhibitory phosphorylation at T495 in eNOS and Y529 in SFK in DMSO-treated cells was not seen in HUVECs treated with SHP099 (Figures S4D–S4F). Interestingly, acute inhibition of SHP2’s activity by SHP099 treatment did not affect VEGFR2 Y1175 or PLCγ phosphorylation (Figures S4D, S4G, and S4H), which was seen by complete SHP2 knockdown (Figures 1K, 1N, S1I, and S1J). As seen for SHP2 downregulation (Figures S1I and S1L), SHP2 inhibition resulted in a trend toward lower VEGFA-stimulated pAKT activation (Figures S4D and S4I). The efficiency of the inhibitor was assessed by blotting for MAPK activation, as previously described^32^ (Figures S4D and S4J). These results, combined with the reduced SHP2 phosphorylation after knockdown of PLCγ *in vitro* (Figures 3E–3I) and *in vivo* (Figures 4A and 4B), suggest that SHP2 might have a dual role in regulating VEGFA-induced vascular permeability, where both scaffolding as well as enzymatic activity are of importance.

The function of SHP2 in regulating the EC barrier was further explored *in vivo* by intradermally injecting 50 μM SHP099 or DMSO control in the back skin of C57BL/6 WT mice, 30 min prior to VEGFA or PBS injection at the same spot. Immunostaining of the skin revealed that VEGFA administration increased the vascular levels of pVEC Y685 in DMSO pre-treated control mice, while these levels were significantly reduced in SHP099 pre-treated mice (Figures 4C and 4D). To investigate the consequence of pharmacological SHP2 inhibition on VEGFA-induced vascular leakage, the Miles assay was employed, analyzing intradermal leakage of the albumin-bound colloidal dye Evans blue. Intradermal administration of VEGFA in control mice, pre-injected with DMSO, induced leakage of Evans blue by approximately 3-fold (Figures 4E and 4F). Compared with the control-treated mice, leakage of Evans blue into the extravascular space was significantly reduced in the mice treated with SHP099 30 min prior to VEGFA injection (Figures 4E and 4F).

In summary, we have identified SHP2 as an additional key player of the pY1175/PLCγ/eNOS/Src pathway. The interplay between SHP2 and PLCγ is essential for VEC Y685 phosphorylation, dismantling of adherens junctions, and vascular leakage in the healthy endothelium.

### VEGFR2 Y1173 heterozygosity is accompanied by decreased tumor endothelial PLCγ/SHP2 signaling and tumor vascular leakage

**Figure 5:**
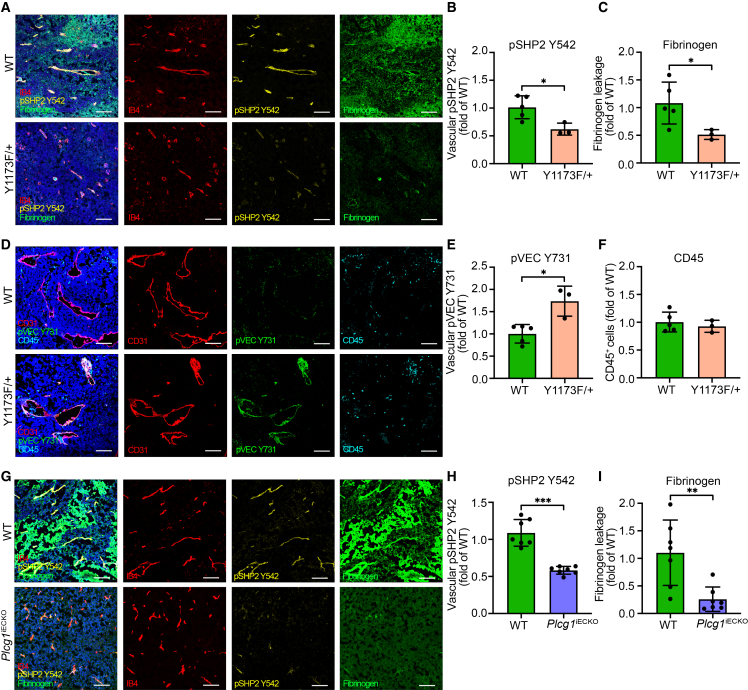
VEGFR2 Y1173 heterozygosity is accompanied by decreased tumor endothelial PLCγ/SHP2 signaling and tumor vascular leakage (A) Representative immunostaining images of *Vegfr2*^*+/+*^ (WT) and *Vegfr2*^*Y1173F/+*^ (Y1173F/+) B16F10 melanoma tumors, showing vessels (IB4; red), pSHP2 Y542 (yellow), and fibrinogen (green). (B and C) Quantification of MFI from (A), shown as fold change over WT. (B) MFI for pSHP2 Y542. (C) MFI for fibrinogen; *n* = 5 (WT) and 3 (Y1173F/+) mice, ≥3 fields of view/mouse. (D) Representative immunostaining images of *Vegfr2*^*+/+*^ (WT) and *Vegfr2*^*Y1173F/+*^ (Y1173F/+) B16F10 melanoma tumors, showing vessels (CD31; red), pVEC Y731 (green), and CD45 (cyan). (E and F) Quantification of MFI from (D), displayed as fold change of WT. (E) MFI for pVEC Y731. (F) MFI for CD45^+^ cells (F); *n* = 5 (WT) and 3 (Y1173F/+), ≥3 fields of view/mouse. Scale bars: 100 μm. (G) Representative immunostaining images of WT and *Plcg1*^*iECKO*^ B16F10 melanoma tumors, showing vessels (IB4; red), pSHP2 Y542 (yellow), and fibrinogen (green). (H and I) MFI quantifications from (G), shown as fold change of WT. (H) MFI for pSHP2 Y542. (I) MFI for fibrinogen; *n* = 7 (WT) and 7 (*Plcg1*^*iECKO*^), ≥3 fields of view/mouse. Unpaired 2-tailed Student’s *t* test. Data represent the mean ± SD. ∗, *p* < 0.05; ∗∗, *p* < 0.01; ∗∗∗, *p* < 0.001. MFI, mean fluorescence intensity. See also Figure S5.

Next, PLCγ/SHP2 signaling was analyzed in the diseased endothelium in experimental tumor models *in vivo*. Immunostaining for pSHP2 Y542, together with fibrinogen and IB4, was performed to assess the correlation between tumor vascular signaling and leakage in the B16F10 melanoma vascular bed. WT mice exhibited activation of pSHP2 Y542 in the tumor endothelium, together with high leakage of fibrinogen (Figures 5A–5C). Diminished pY1173/PLCγ signaling in B16F10 *Vegfr2*^*Y1173F/+*^ tumors instead resulted in reduced endothelial SHP2 signaling and fibrinogen leakage from tumor vessels (Figures 5A–5C).

SHP2 has recently been described to dephosphorylate VEC at the pY731 site, in response to immune cell adhesion, mediated by PECAM-1 (CD31) signaling.^17^ We, therefore, aimed to analyze whether pVEC Y731 levels were affected upon alteration of SHP2 signaling *in vivo*. Evidently, B16F10 tumors from *Vegfr2*^*Y1173F/+*^ mice exhibited enhanced vascular pVEC Y731 levels as compared to WT tumors, in accordance with the identified role of SHP2 in dephosphorylating pVEC Y731 (Figures 5D and 5E). Co-stainings for CD45, a general immune cell marker, did not reveal a difference in the intratumoral composition between WT and *Vegfr2*^*Y1173F/+*^ tumors (Figures 5D and 5F), also in line with previously described results in this tumor model.^19^ However, Y731 phosphorylation might still contribute to the altered recruitment and activation of specific immune cell subsets.^16^^,^^17^ To assess if the altered pVEC Y731 signaling was a direct consequence of lower pVEGFR2 Y1175 signaling, HUVECs were treated with *siCtr* or *siPTPN11* and stained for pVEC Y731 subsequent to VEGFA treatment. In control-treated cells, VEGFA stimulation did not affect junctional pVEC Y731 levels, suggesting a regulation independent of VEGFA signaling (Figures S5A and S5B). However, downregulation of SHP2 did result in a minor, although not significant, VEGFA-stimulated increase in junctional pVEC Y731 (Figures S5A and S5B). The impact of the additional interaction partners, CSK and VAV2, was also analyzed. An increase in pVEC Y731 signaling following CSK downregulation occurred independently of VEGFA stimulation (Figures S5C and S5D), in line with CSK’s established role in terminating SFK signaling and reducing VEC phosphorylation.^27^^,^^29^ Notably, downregulation of VAV2 did not alter endothelial pVEC Y731 levels (Figures S5C and S5D).

The direct effect of PLCγ in enhancing VEGFA-induced normal vascular permeability was previously demonstrated by assessing vascular leakage in the back skin of C57BL/6 mice with an inducible knockdown of PLCγ in the endothelium (*Plcg1*^*iECKO*^ mice).^19^ To extend these findings and confirm a direct effect of PLCγ in mediating vascular leakage in tumor vessels, B16F10 melanoma tumor cells were inoculated into the flank of *Plcg1*^*iECKO*^ mice or WT control mice. The tumors were allowed to grow for 13 days, and 3 h prior to tumor dissection, the mice were intravenously injected with a 70 kDa TRITC dextran. The results showed no difference in the tumor growth rates (Figure S5E) or vessel density (Figure S5F) between the groups. However, *Plcg1*^*iECKO*^ mice had reduced leakage of 70 KDa TRITC dextran (Figure S5G) into the tumor microenvironment, evaluated by solvent-based extraction of dextran, as previously described.^19^ Immunostainings of tumor tissue showed lower phosphorylation of pSHP2 Y542 in the tumor endothelium, as well as reduced fibrinogen leakage in *Plcg1*^*iECKO*^ mice, as compared to WT littermate control mice (Figures 5G–5I).

### Tumor vessel expression of PLCγ and SHP2 correlates with vascular leakage in human cancers

The identified mechanistic findings of PLCγ/SHP2 signaling-induced VEC opening and vascular permeability downstream of pVEGFR2 Y1173 in the diseased endothelium prompted investigation of the potential clinical relevance. Our recent investigations of endothelial PLCγ expression in human cancers revealed prominent expression in several cancers, and in a subgroup of kidney cancer patients, vascular PLCγ was identified as a biomarker for a poor patient prognosis.^19^ In this study, the clinical relevance of PLCγ/SHP2 signaling and assessment of vascular leakage in human cancer were further examined in patient biopsies.

Tumor tissue expression of SHP2 was initially analyzed by immunohistochemistry (IHC) stainings from the Human Protein Atlas consortium.^34^^,^^35^ In 7 randomly selected cancer forms, including bladder, breast, liver, ovarian, and kidney (renal cell carcinoma: RCC) cancers and glioma and melanoma, SHP2 expression was found in the tumor vasculature (Figures S6A and S6B). Similar to what we previously identified for PLCγ,^19^ out of all cancer forms analyzed, SHP2 was almost exclusively expressed in the vasculature only in RCC (Figures S6A and S6B). Analysis of SHP2 levels in RCC further identified that a subgroup of patients had lost the expression in the tumor vasculature (Figure S6B, upper left panel). Consecutive sections stained for the vessel marker CD31 showed similar vessel density between patients (Figure S6B, lower panel). To further explore the tumor vascular levels in RCC, we performed immunofluorescent co-stainings of SHP2 together with the vessel marker CD34. We confirmed tumor endothelial SHP2 expression in bladder cancer and two additional cancers, namely testicular and stomach cancer (gastrointestinal adenocarcinoma, GAC) (Figure S6C). A similar pattern was seen in RCC where 6 out of 16 patients had low SHP2 expression in tumor vessels (SHP2 low), and the remaining 10 patients presented with high expression levels (SHP2 high) (Figure S6D).

**Figure 6:**
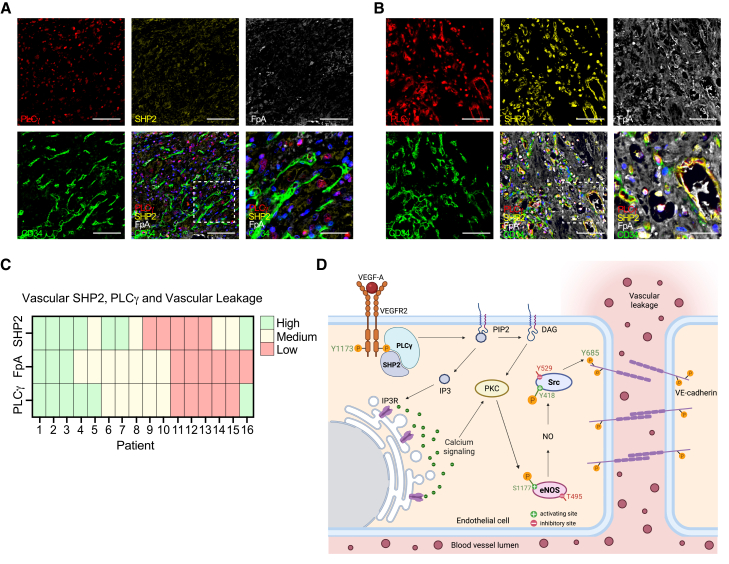
SHP2 and PLCγ are clinically relevant biomarkers for tumor vascular leakage (A and B) Representative multiplex images of RCC patient biopsies. (A) Multiplex images for RCC patients with low vascular PLCγ and SHP2 expression. (B) Multiplex images for RCC patients with high vascular PLCγ and SHP2 expression. Tissue sections were stained for PLCγ (red), SHP2 (yellow), CD34 (green), and FpA (white) to visualize vascular leakage, and counterstained with Hoesht. Scale bars: 100 μm. Boxed regions are shown at higher magnification to the right. Scale bars: 30 μm. (C) Heatmap comparing endothelial SHP2 expression, PLCγ expression, and vascular leakage scores across individual RCC patients (*n* = 16). Patients are arranged by their ID number, with color coding indicating high (green), medium (yellow), or low (red) levels. (D) Schematic for the mechanism of SHP2/PLCγ interaction upon VEGFR2 activation. In summary, VEGFA stimuli leads to the phosphorylation of VEGFR2 Y1175, which recruits both PLCγ and SHP2. SHP2 is needed for the activation of PLCγ at the plasma membrane and hydrolysis of PIP2 to IP3 and DAG. The former triggers the release of intracellular calcium (Ca^2+^), and together with DAG, activates PKC, causing phosphorylation of the eNOS activating site S1177 and dephosphorylation of the inactivating site T495. Production of NO mediates nitration and full activation of Src, which phosphorylates Y685 of VEC, disrupting the adherens junctions and resulting in vascular leakage. Created in BioRender. Kremmyda, P. (2025). RCC, renal cell carcinoma; FpA, fibrinopeptide A; PIP2, phosphatidylinositol-4,5-bisphosphate; IP3, inositol-1,4,5-trisphosphate; DAG, diacylglycerol; PKC, protein kinase C; eNOS, endothelial nitric oxide synthase; NO, nitric oxide; VEC, VE-cadherin. See also Figure S6.

To assess the correlation among tumor vascular SHP2, PLCγ, and vascular leakage, multiplex immunostainings were employed. In addition to antibodies against SHP2 and PLCγ, antibodies detecting fibrinopeptide-A (FpA) were used to identify vascular leakage, as well as CD34 as a vessel marker (Figures 6A and 6B). FpA is a 16-amino acid short peptide within the fibrinogen Aa chain that gets extravasated from the blood vasculature upon ligand (including VEGFA)-stimulated barrier breakdown, which has been validated as a surrogate marker for tumor vessel leakage.^36^ The results confirmed findings of prominent but variable PLCγ expression^19^ and SHP2 expression in the endothelium of RCC patients, where certain patients had lost the vascular expression (Figure 6A). Six out of 16 examined patient biopsies had high levels of PLCγ in the endothelium, 5 showed medium levels, and 5 patients showed low levels (Figures 6A–6C). A relative quantification of vascular SHP2 into low, medium, and high levels revealed 5 patients with low, 4 patients with medium, and 7 patients with high levels (Figure 6C). The extent of FpA staining in the extravascular space was quantified to assess vascular leakage. Relative quantification showed 3 patients with high leakage, 7 patients with medium leakage, and 6 patients with low leakage (Figure 6C). In accordance with what has previously been seen in healthy vessels,^36^ some patients with low levels of FpA in the microenvironment instead displayed positive staining within the vessel lumen (Figure 6A). Stainings showed that patients with low vascular PLCγ levels exhibited low FpA leakage, and patients with high PLCγ levels showed high leakage of FpA (Figures 6A–6C). In line with this observation, statistical analysis revealed a significant association between vascular PLCγ levels and vascular leakage of FpA (χ^2^ test, *p* = 0.004). Moreover, the results showed that patients with high vascular SHP2 also had high levels of FpA signal in the extravascular space (Figure 6B). A significant correlation between SHP2 and tumor vascular leakage was also found (χ^2^ test, *p* = 0.037). Interestingly, most patients with high levels of endothelial SHP2 also showed high levels of endothelial PLCγ, further supporting their interplay *in vivo*.

In summary, SHP2 exerts regulatory functions on VEGFR2 Y1175/PLCγ signaling in tumor endothelial cells, which impairs the vascular barrier, resulting in increased leakage in kidney cancer patients.

## Discussion

This study extends previous work^19^ and explores the molecular mechanism by which VEGFR2 Y1175/PLCγ signaling enhances macromolecular leakage. Out of four potential candidates previously captured in an MS screen^19^—RASA1, CSK, VAV2, and SHP2—SHP2 was here identified as an important key regulator. SHP2 was shown to interact with PLCγ at pY1175, and the interplay between them was crucial for downstream activation of eNOS and Src, leading to subsequent phosphorylation of VEC Y685 and disintegration of endothelial junctions (Figure 6D). The VEGFR2 Y1175/PLCγ/SHP2 pathway was demonstrated as a clinically relevant pathway mediating VEGFA-induced barrier destabilization and vascular leakage both in healthy and tumor vasculature.

The non-receptor protein tyrosine phosphatase SHP2 is indispensable for normal vascular development, as SHP2 gene deletion in endothelial and vascular smooth muscle cells leads to vascular defects and embryonic lethality in mice.^37^^,^^38^ In both human cancer and mouse tumor models, SHP2 has been reported to be upregulated in tumor endothelial cells compared to normal endothelium, suggesting SHP2 to be a relevant tumor vascular target, with minor effects on normal endothelial cell functions upon inhibition.^39^^,^^40^ Normal organ vasculature also remained structurally and functionally intact after genetic or pharmacological targeting of SHP2 in adult mice.^39^^,^^40^ Consistent with these studies, a subset of RCC patients expressed elevated endothelial SHP2 and PLCγ, which significantly correlated with increased vascular leakage, highlighting the VEGFR2 pY1175/PLCγ/SHP2 axis as clinically relevant for vascular dysfunction (Figures 6A–6C) and supporting previously reported vascular PLCγ as a biomarker for poor patient prognosis.^19^

The role of this pathway in regulating macromolecular leakage was confirmed *in vivo* by using a B16F10 melanoma mouse tumor model. Tumors with defective VEGFR2 Y1173/PLCγ signaling from *Vegfr2*^*Y1173F/+*^ mice exhibited significantly decreased endothelial SHP2 phosphorylation, accompanied by reduced fibrinogen leakage (Figures 5A–5C). Moreover, endothelial-specific deletion of PLCγ (*Plcg1*^*iECKO*^) or pharmacological inhibition of SHP2 (SHP099) suppressed VEGFA-induced signaling and vascular leakage *in vivo*, both in normal and tumor vessels (Figures 4A–4F and 5G–5I). These findings align with those of recent studies demonstrating that inducible endothelial deletion of SHP2 (*Ptpn11*^*iECKO*^) or pharmacological inhibition of SHP2 decreased tumor neovascularization, though with different outcomes on the EC barrier. *Ptpn11*^*iECKO*^ mice exhibited tumor vessel normalization with reduced leakage, while the mice treated with SHP2 inhibitors showed enhanced leakage as a result of endothelial cell apoptosis.^39^^,^^40^ In line with the latter, during normal embryonic development, genetic deletion of SHP2 resulted in enhanced vessel hemorrhage due to VEC internalization mediated by MET-ARF1 signaling,^37^ suggesting that various outcomes might differ between the developing vasculature and already established vessels.^37^^,^^39^^,^^40^

The novelty of the current study is the identification of SHP2’s role downstream of VEGFR2 in promoting vascular permeability *in vivo*, both in normal and tumor tissues. There are reports showing that SHP2 affects tumor permeability through other pathways, independent of VEGFR2, although there is limited evidence *in vivo*. Mechanisms have been proposed for SHP2’s involvement in promoting cell survival and tumor angiogenesis, including suppression of the pro-apoptotic STAT3 cascade, as well as the proliferative MAPK cascade.^39^ Moreover, a recent report by Xu et al. demonstrated that SHP2 dephosphorylates ASK1, followed by c-Jun activation and upregulation of the pro-angiogenic transcription factor SOX7.^40^ This contributed to leaky, immature vessels with a poor pericyte coverage. In our experimental models, VEGFR2 pY1173/PLCγ/SHP2 signaling mediated vascular leakage without affecting tumor growth or vessel density (Figures S5E and S5F). Nevertheless, we cannot ignore that findings are dependent on the tumor model selected. There might also be organotypic differences involved, previously described for VEGFR2 signaling by Karaman et al.^41^ The effect of SHP2 inhibitors on endothelial cells in already established tumors, which is a more relevant approach for clinical evaluation, has not yet been explored. Targeting SHP2 might inhibit tumor angiogenesis at an early stage when vessel remodeling is high, but lead to vessel normalization by impairing pY1175/PLCγ signaling at a later stage. The opposing outcomes on EC barrier stability and tumor vascular normalization between genetic deletion and pharmacological targeting might also be caused by indirect effects of the inhibitors.

The role of individual VEC tyrosine residues has been reported in studies on Y685F- and Y731F-mutant knock-in mice.^16^^,^^42^ VEC Y685 phosphorylation induced vascular leakage, and pVEC Y731 selectively controlled leukocyte extravasation.^16^ Upon leukocyte engagement, SHP2 was released from PECAM-1 (CD31) to dephosphorylate VEC at Y731 and enable diapedesis.^16^^,^^17^ On the contrary, Y685F mice had a minor but significant increase in transmigration of neutrophils in cremaster tissue relative to WT mice.^16^ Recently, the mechanism of how pY685 counteracts leukocyte diapedesis, via cortactin and ICAM1, was revealed.^28^ In contrast to pVEC Y685, which gets phosphorylated in response to VEGFA, pVEC Y731 was not affected by VEGFA stimulation *in vitro* and *in vivo*.^16^^,^^19^ This is in line with observations in the current study; VEGFA stimulation of HUVECs increased pVEC Y685 but had no effect on Y731. Furthermore, knockdown of SHP2 significantly prevented VEGFA-induced pY685 (Figures 1C–1E) and only slightly enhanced pY731 (Figures S5A and S5B). In concordance, Arif et al. showed that the overexpression of SHP2 in HUVECs did not lead to dephosphorylation of Y731 and that mechanical cues are also needed to unmask pY731 from catenins.^17^ According to the findings, SHP2 might exhibit a dual function in regulating endothelial junctions, which might be dependent on its cellular localization in response to different stimuli. In resting endothelial cells, SHP2 interacts with PECAM-1 (CD31), and upon leukocyte adhesion, SHP2 gets localized to VEC to dephosphorylate Y731, leading to VEC internalization and immune cell passage across the vessel wall.^17^ Instead, in response to high VEGFA levels, SHP2 gets recruited to pVEGFR2 Y1175 to activate downstream eNOS/Src, resulting in VEC Y685 phosphorylation, endothelial junctional disruption, and vascular leakage. We did identify altered cellular localization of SHP2 and SHP2/PLCγ complexes upon VEGFA stimulation *in vitro* (Figures S1G–S1H and S3K–S3L). However, for detailed alteration of SHP2’s localization to individual molecular mediators, high-resolution imaging is encouraged. The regulation of SHP2 *in vivo* is most likely more complex. We saw a prominent increase in vascular pVEC Y731 in our tumor models, with repressed PLCγ/SHP2 signaling (Figures 5D and 5E). This is likely a result of an altered tumor immune microenvironment and not a direct effect of diminished pVEGFR2 Y1173 signaling. If enhanced pVEC Y731 contributes to the observed changes in immune cell infiltration in *Vegfr2*^*Y1173F/+*^ tumors is an area for exploration in continued studies.

By an unbiased approach, we have identified that SHP2 interacts with the pY1175 phosphosite in VEGFR2. An association of SHP2 with VEGFR2 *in vitro* has previously been described.^43^^,^^44^^,^^45^ Studies conducted in cultured endothelial cells suggest roles for SHP2 in receptor dephosphorylation and internalization^43^ or in promoting MAPK signaling without affecting internalization of RTKs.^44^ In concordance, our results did not indicate the involvement of SHP2 in VEGFR2 internalization. Instead, downregulation of SHP2 decreased pVEGFR2 Y1175 levels upon VEGFA stimulation, with no effect on the total VEGFR2 levels (Figures 1K and 1N). This was not seen upon SHP099 treatment (Figures S4D and S4G), suggesting that absence of the SHP2 protein itself, and not necessarily SHP2 activation, was responsible for lower pVEGFR2 Y1175 and pPLCγ levels.

SHP2 has two SH2 domains, a catalytic PTP domain and two C-terminal tyrosine phosphorylation sites (pY542 and pY580). The N-terminal SH2 domain (N-SH2) blocks the catalytic PTP domain, keeping SHP2 enzymatically inactive. Binding of SHP2 to tyrosine residues releases this autoinhibition and promotes enzymatic activation.^46^^,^^47^ Both phosphatase-dependent and -independent functions of SHP2 have been described in the literature.^30^^,^^31^^,^^48^ A recent report showed that PLCγ and SHP2 bind directly through heterodimerization of their tandem SH2 domains in a phosphorylation-independent manner.^31^ Authors further showed that SHP2 and PLCγ form a ternary complex with fibroblast growth factor receptor 2 (FGFR2), assembled upon receptor phosphorylation. The pY769 of FGFR2 recruited SHP2 to form a scaffold for phosphorylated PLCγ, which ensured access of active PLCγ to its phospholipid substrates.^31^ The importance of SHP2’s scaffolding function was shown by the expression of a phosphatase-dead mutant (SHP2C459S) that restored downstream signaling in SHP2-knockout cells.^31^ In accordance, Cruz-Duarte et al. found an interaction of SHP2 and PLCγ and described their interplay, required for Ras activation.^30^ Our results primarily suggest that upon VEGFA binding to VEGFR2, SHP2 gets recruited to pY1175, forms a complex with PLCγ and acts as a scaffold to induce downstream signaling and vascular leakage. In addition to this phosphatase-independent role, interaction with PLCγ at pY1175 also enhances SHP2 Y542 and Y580 phosphorylation, which is not predominantly needed for activation but has been suggested to stabilize the active confirmation and enhance scaffolding functions.^49^^,^^50^ This is evident by reduced pSHP2 levels *in vivo* in vessels with aberrant PLCγ signaling (Figures 4A, 4B, 5A–5C, and 5G–5I), as well as *in vitro* in ECs with knockdown of PLCγ (Figures 3E–3I and S3F–S3J).

When SHP2 acts as a scaffold for PLCγ, each protein’s tandem SH2 domains should be free to interact. SHP099 is an allosteric inhibitor of SHP2 that binds to a surface other than the active site, inducing a conformational change, which restricts the enzymatic activity. Here, we showed that SHP099 treatment reduced VEGFA-induced signaling and vascular leakage *in vivo* (Figures 4C–4F) and *in vitro* (Figures S4A–S4J). When SHP2 is inhibited by SHP099, the N-SH2, C-SH2, and PTP domains are “glued” together in a closed conformation, suggesting that PLCγ-scaffolding is also impaired. In line with this assumption, although siRNA-mediated knockdown of SHP2 and pharmacological inhibition both suppressed endothelial VEGFA-induced activation of eNOS and Src (Figures 1K–1M, 2A–2H, 3A–3D, and S4A–S4C), pharmacological targeting still resulted in PLCγ phosphorylation (Figures S4D and S4H). This suggests that instead of affecting PLCγ phosphorylation, SHP099 blocks the recruitment of PLCγ to the endothelial cell membrane. This was further supported by the absence of MAPK activation subsequent to SHP2 blockade (Figures S4D and S4J). Whether SHP099 limits access of PLCγ to its substrate phosphatidylinositol 4,5-biphosphate and diminishes downstream Ca^2+^ release from the ER currently remains unknown. Notably, although not significant, we did observe a reduction in PI3K/AKT signaling upon SHP2 knockdown (Figures S1I and S1L) and pharmacological inhibition *in vitro* (Figures S4D and S4I). Thus, the reduced PLCγ signaling could also be affected by reduced PI3K production of phosphatidylinositol-3,4,5-trisphosphate (PIP3), which is involved in anchoring PLCγ to the plasma membrane.^51^

To further elucidate the phosphatase-dependent and -independent roles of SHP2 in endothelial cells, future studies are warranted, and development of phosphatase-independent inhibitors is required to obtain a more detailed mechanistic insight. Our study supports SHP2’s involvement in the development of an abnormal, hyperpermeable vasculature that forms during tumor angiogenesis and prompts further development of pharmacological inhibitors against SHP2 as an anti-angiogenic cancer and vascular normalization therapy.

### Limitations of the study

Our work provide evidence of SHP2’s involvement in VEGFR2 Y1175-induced vascular permeability, in normal and tumor vasculature. However, there are limitations that needs to be acknowledged. The current study focuses on VEGFA stimulated activation of PLCγ/SHP2, but there are also additional pathways and downstream activators known to activate vascular leakage. The phosphatase dependent and independent roles of SHP2 in mediating vascular permeability are not fully elucidated in the current study, and would need further exploration. The mechanistic data is discovered by experiments *in vitro* (in HUVECs) and in mouse models, and might not entirely recapitulate human tumor vasculature and organotypic differences. Additionally, the selected tumor model (B16F10 melanoma) is a commonly used syngeneic model, but results would be further strengthened by analysis of additional tumor models. Also, experiments on how SHP2 knockout or chemical inhibition affects tumor growth, survival and treatment response, downstream of Y1173/PLCγ signaling, would further strengthen the clinical relevance of our work. The small number of patients (16) is also a limitation and correlation to tumor leakage needs validation in larger cohorts with survival data, to confirm the clinical importance.

## Resource availability

### Lead contact

Requests for further information and resources should be directed to and will be fulfilled by the lead contact, Elin Sjöberg (elin.sjoberg@igp.uu.se).

### Materials availability

This study did not generate new unique reagents.

### Data and code availability

• Mass spectrometry data were originally published in Sjöberg et al.^19^ and are available via ProteomeXchange with identifier PXD041024, as well as listed in key resources table.
• This paper does not report any original code.
• Any additional information required to reanalyze the data reported in this paper is available from the lead contact upon request.

## Acknowledgments

This work is supported by funding from the 10.13039/100007435Åke Wiberg Foundation (M22–0141, M23–0182, and M24-0246), 10.13039/501100006285Magnus Bergvall Foundation (2021-04476, 2022-388, 2023-719, and 2024-1266), the 10.13039/501100003748Swedish Society for Medical Research (10.13039/501100003748SSMF) (P17-0144), and 10.13039/501100004063Knut and Alice Wallenberg Foundation (KAW 2020.0057). The authors acknowledge Prof. Lena Claesson-Welsh (Department of Immunology, Genetics and Pathology, Uppsala University) for valuable scientific input during the course of the project and Pernilla Martinsson, Dr. Emmanuel Nwadozi, and Dr. Yindi Ding at the Department of Immunology, Genetics and Pathology, Uppsala University, for technical skills and experimental expertise. The following mice strains are originally kind gifts: *Plcg1*^*fl/fl*^ from Dr. Renren Wen (10.13039/100032233Versiti Blood Research Institute, Milwaukee, Wisconsin USA),^52^ Cdh5(PAC)-CreERT from Prof. Ralf Adams (Department of Tissue Morphogenesis, Max Planck Institute for Molecular Biomedicine, Münster, Germany), and *Vegfr2*^*Y1173*^ from Prof. Masabumi Shibuya (Institute of Physiology and Medicine, Jobu University, Takasaki, Gunma, Japan). The authors also thank Prof. Mike Simons and Dr. Dongying Chen (Yale Cardiovascular Research Center, 10.13039/100017094Department of Internal Medicine, Yale University School of Medicine, New Haven, Connecticut, USA) for crossing the *Plcg1*^*fl/fl*^ mice with Cdh5(PAC)-CreERT to generate the *Plcg1*^*iECKO*^ strain.

## Author contributions

P.K. and E.S. conceptualized the project; P.K., S.O., S.P., E.W., C.C., and E.S. developed the methodology; P.K., S.O., S.P., E.W., C.C., C.L., and E.S. conducted experiments and interpreted data; C.L. and E.S. acquired funding for the project; P.K. and E.S. drafted the manuscript. All authors reviewed and edited the manuscript.

## Declaration of interests

The authors declare no competing interests.

## STAR★Methods

### Key resources table

**Table:** 

REAGENT or RESOURCE	SOURCE	IDENTIFIER
**Antibodies**		

Rabbit anti-phospho-Akt (Ser473)	Cell Signaling Technology	4058S; RRID: AB_331168
Rabbit anti-Akt	Cell Signaling Technology	9272S; RRID: AB_329827
Goat anti-CD31	R&D Systems	AF3628; RRID: AB_2161028
Mouse anti-human CD34	DAKO	M7165; RRID: AB_2063006
Rat anti-mouse CD45	BD Pharmingen	553076; RRID: AB_394606
Rabbit anti-CSK	Cell Signaling Technology	4980S; RRID: AB_2276592
Mouse anti-CSK	Invitrogen Thermo Fisher	MA5-15707; RRID: AB_10981163
Mouse anti-phospho-eNOS (S1177)	BD Pharmingen	612393; RRID: AB_399751
Mouse anti-phospho-eNOS (T495)	BD Pharmingen	612707; RRID: AB_399946
Mouse anti-eNOS	BD Pharmingen	610296; RRID: AB_397690
Rabbit anti-phospho-ERK1/2	Cell Signaling Technology	9101S; RRID: AB_331646
Mouse anti-ERK	Cell Signaling Technology	4696S; RRID: AB_390780
Goat anti-mouse fibrinogen	Nordic-MUbio	GAM/Fbg/7S; RRID: AB_3719479
Mouse anti-human fibrinopeptide A	Novus	NB100-73043; RRID: AB_1108550
Mouse anti-GAPDH	Merck	MAB374; RRID: AB_2107445
Alexa Fluor 594–Isolectin GS-IB4	Thermo Fisher Scientific	I21413; RRID: AB_2313921
Rabbit anti–phospho-PLCγ1 (Tyr783)	Cell Signaling Technology	2821S; RRID: AB_330855
Rabbit anti-PLCγ1	Cell Signaling Technology	2822S; RRID: AB_2163702
Mouse anti-PLCγ (E−12)	Santa Cruz Biotechnology	sc-7290; RRID: AB_628119
Mouse anti-RASA1	Thermo Fisher Scientific	MA4-001; RRID: AB_2175873
Rabbit anti–phospho-SHP2 (Y542)	Cell Signaling Technology	3751S; RRID: AB_330825
Rabbit anti–phospho-SHP2 (Y580)	Cell Signaling Technology	3703S; RRID: AB_2174962
Rabbit anti-SHP2 (DSOF2)	Cell Signaling Technology	3397S; RRID: AB_2174959
Mouse anti-PTPN11 (SHP2)	Origene	TA501914; RRID: AB_11126900
Mouse anti-SH-PTP2	Santa Cruz Biotechnology	sc-7384; RRID: AB_628252
Rabbit anti-phospho-Src (Y416)	Cell Signaling Technology	2101S; RRID: AB_331697
Rabbit anti-phospho-Src (Y527)	Cell Signaling Technology	2105S; RRID: AB_331034
Rabbit anti-Src	Cell Signaling Technology	2123S; RRID: AB_2106047
Mouse anti-Src	Abcam	ab231081; RRID: AB_2917962
Mouse anti-VAV2	Invitrogen Thermo Fisher	MA5-38657; RRID: AB_2898569
Rabbit anti-VAV2	Cell Signaling Technology	2848S; RRID: AB_2213746
Goat anti-VE-cadherin	R&D Systems	AF1002; RRID: AB_2077789
Rabbit anti-VE-Cadherin pY685	In-house	Jin et al.^29^
Rabbit anti-VE-Cadherin pY731	In-house	Jin et al.^29^
Rabbit anti-phospho-VEGFR2 (Tyr1175)	Cell Signaling Technology	2478S; RRID: AB_331377
Rabbit anti-VEGFR2	Cell Signaling Technology	2479S; RRID: AB_2212507
Mouse anti-Yes	BD Biosciences	610376; RRID: AB_397759
Mouse anti-3-nitrotyrosine	Abcam	ab61392; RRID: AB_942087
Amersham ECL Rabbit IgG, HRP-linked	Cytiva	NA934; RRID: AB_772206
Amersham ECL Mouse IgG, HRP-linked	Cytiva	NA931; RRID: AB_772210

**Biological samples**		

Human tumor tissue microarray (TMA)	Human Protein Atlas (HPA)	Uhlén et al.^35^

**Chemicals, peptides, and recombinant proteins**		

VEGFA 165	Peprotech	450–32
SHP099	MedChemExpress	HY-100388
Endothelial Cell Growth Medium MV2	PromoCell	C-39221
Opti-MEM Medium	Gibco	31985062
Lipofectamine RNAiMAX	Thermo Fisher Scientific	13778075
DMEM, high glucose, GlutaMAX™ Supplement, pyruvate	Gibco	10569–010
Pyrilamine maleate salt	Sigma	P5514
70 kDa TRITC dextran	Tdb labs	TD70
DAPI Fluoromount-G®	SouthernBiotech	0100–20

**Critical commercial assays**		

Duolink® *In Situ* Red Starter Kit Mouse/Rabbit	Duolink	DUO92101
NaveniFlex™ Cell MR	Navinci	NC.MR.100
BLOXALL® Endogenous Blocking Solution	Vector Labs	SP-6000-100
ImmPRESS-VR horse anti-mouse IgG	Vector Labs	MP-6402-15
ImmPRESS-VR horse anti-rabbit IgG	Vector Labs	MP-7801-15
Opal 480 Reagent Pack	Akoya Biosciences	FP1500001KT
Opal 520 Reagent Pack	Akoya Biosciences	FP1487001KT
Opal 570 Reagent Pack	Akoya Biosciences	FP1488001KT
Opal 620 Reagent Pack	Akoya Biosciences	FP1495001KT
Opal 690 Reagent Pack	Akoya Biosciences	FP1497001KT
Opal 780 Reagent Pack	Akoya Biosciences	FP1501001KT
1X Plus Automation Amplification Diluent	Akoya Biosciences	FP1609
Serum-free protein blocking buffer	DAKO	X0909
Target retrieval solution, citrate pH6.1	DAKO	S1699

**Deposited data**		

Mass spectrometry data (VEGFR2 pY1175 interactome)	Sjöberg et al.^19^	ProteomeXchange with identifier PXD041024

**Experimental models: Cell lines**		

Human Umbilical Vein Endothelial Cells (HUVEC)	PromoCell	C-12203
B16F10	American Type Culture Collection	CRL-6475; RRID:CVCL_A4CJ

**Experimental models: Organisms/strains**		

C57Bl/6 mice	Taconic Biosciences	B6JBOM; RRID: IMSR_TAC:B6JBOM
*Vegfr2*^*Y1173F*^ mice	Institute of Physiology and Medicine, Jobu University, Takasaki, Gunma, Japan	Sakurai et al.^23^
*Plcg1*^*fl/fl*^ mice	Versiti Blood Research Institute, Milwaukee, Wisconsin, USA	Fu et al.^52^
*Plcg1*^*iECKO*^ mice	Yale Cardiovascular Research Center, Department of Internal Medicine, Yale University School of Medicine, New Haven, Connecticut, USA	Sjöberg et al.^19^

**Oligonucleotides**		

*siCSK*	Merck	SASI_Hs01_00097531
*siPLCG1*	Merck	SASI-Hs01-0020-7375
*siPTPN11*	Merck	SASI_Hs01_00207375
*siVAV2*	Merck	SASI_Hs01_00118656
*Silencer*™ Select Negative Control No. 1 siRNA	Invitrogen™	4390843

**Software and algorithms**		

GraphPad Prism 10	GraphPad Software Inc.	RRID:SCR_002798
Fiji/ImageJ	https://imagej.nih.gov/ij/download.html	RRID:SCR_003070
Adobe Illustrator	Adobe Inc.	RRID:SCR_010279

### Experimental model and study participant details

#### Animals

The generation of *Vegfr2*^*Y1173F*^ and *Plcg1*^*fl/fl*^ mice has been described in detail.^23^^,^^52^ Moreover, *Plcg1*^*iECKO*^ mice were generated by crossing with the Cdh5(PAC)-CreERT2 strain, as previously described.^19^ Mice were housed in ventilated cages (2–5 mice per cage) and maintained through heterozygous or homozygous matings. Tamoxifen (Merck) was administered intraperitoneally at a dosage of 80 mg/kg for five consecutive days to initiate Cre recombinase-mediated gene recombination. Following this treatment, the mice were given a 7-day recovery period before experiments were performed. Wild-type C57Bl/6 mice were purchased from Taconic Biosciences and used for the Miles Assay. A minimum of 3 animals per genotype was included in each experiment, representing individual biological replicates. Mice were allocated in experimental groups based on genotype. Mice of 8–10 weeks old were included and the sample size was selected to guarantee reproducibility and ensure stringent statistical analysis. Both male and female mice were used to avoid any impact of gender bias. Data was pooled and no analysis on sex-specific differences was performed. The animal experiments were performed following the Swedish Board of Agriculture guidelines and approved by the regional ethical committee, Uppsala, Sweden (permit 5.8.18–05312/2023).

#### Cell culture

Human umbilical vein endothelial cells (HUVECs) (PromoCell) were cultured in endothelial cell culture medium MV2 (PromoCell, #C-39221) with supplements: FCS-25, hEGF-2.5, HC-100, VEGF-0.25, hbFGF-5, R3 IGF-1, AA-500. Donor-sex information was not provided by the supplier. Cells were maintained at 37°C with 5% CO_2_ and used only at low passages (maximum 6 passages). Cells were seeded in 8-well chamber slides at 5 x 10^4^ cells/well and in 6-well plates at 3.5 x 10^5^ cells/well. Starvation of cells for 3 h with MV2 Medium without supplements was followed by VEGFA165 stimulation at a final concentration of 100 ng/mL (Peprotech, 450-32). SHP2 chemical inhibition was performed by treating cells with 30 μM SHP099 (MedChemExpress, HY-100388) in Dimethyl sulfoxide (DMSO) diluted in starvation medium for 3 h. Starvation media with DMSO was used as the unstimulated control. Cells were stimulated for 5 min at 37°C, except for the VEGFA stimulation timepoints experiments, where cells were stimulated with VEGFA for 2, 5, 10 and 20 min. Stimulation was terminated by washing cells with cold phosphate-buffered saline (PBS) (Gibco, 10010-015). For subcutaneous mouse tumor experiments, melanoma cells B16F10 (American Type Culture Collection) were cultured in DMEM, high-glucose, GlutaMAX Supplement, pyruvate medium (Gibco) with Penicillin/Streptomycin mix (Thermo Fisher Scientific). Cells were regularly tested for mycoplasma infections. Cell lines were newly obtained prior to this study and no authentication of identity was performed.

#### Patient samples

Human tumor tissue microarray (TMA) containing formalin-fixed paraffin-embedded core biopsies of patients with different cancer forms, generated by Human Protein Atlas (HPA) consortium,^34^^,^^35^ were examined, including bladder- (*n* = 12), stomach- (GAC) (*n* = 12), kidney (RCC) (*n* = 16) and testicular cancer (*n* = 12), using immunofluorescent staining. Two core biopsies were collected per patient and samples were sectioned to 4 μm thickness and mounted on Superfrost Plus microscope slides (Thermo Fisher Scientific). Subsequent to immunofluorescent staining, RCC patients (*n* = 16) were scored by two independent investigators and patients were divided into groups based on a consensus relative protein score. Tumor TMA from HPA is covered by the HPA ethical permit (EPN Uppsala, Sweden, 2002/577,2011/473). All patients were anonymous and between 52 and 83 years old. No information on ethnicity or race was available. Both females and males were included and gender distribution had no impact on the study results. Written informed consent from all patients was received prior to study participation. The studies complied with the 1975 Declaration of Helsinki, as revised in 1983.

### Method details

#### RNAi interference

The siRNAs were packaged in Lipofectamine RNAiMAX (Thermo Fisher Scientific, 13778075) and diluted in Opti-MEM Medium (Gibco, 31985062) in a final concentration of 10 nM. Fresh MV2 medium was added the following day, and experiments were performed 48–72 h after siRNA transfection.

#### SDS-PAGE and immunoblotting

Cells were harvested and proteins extracted using RIPA Buffer (Thermo Fisher Scientific, 89901) supplemented with 50 nM Na_3_VO_4_, Phosphatase inhibitor cocktail (Roche, 04906837001) and Protease inhibitor cocktail (Roche, 04693116001). Protein lysates were mixed with LDS sample buffer (Thermo Fisher Scientific, NP0007) and sample reducing agent (Thermo Fisher Scientific, NP0009) before incubation at 97^o^C for 5 min. Protein lysates and immunoprecipitates were separated on NuPAGE 4–12% Bis-Tris gels (Thermo Fisher Scientific) in MOPS SDS running buffer (Thermo Fisher Scientific, NP0001) using PageRuler Plus Prestained protein ladder (Thermo Fisher Scientific, 26619) as molecular weight ladder. Later, proteins were transferred to polyvinylidene difluoride (PVDF) membranes (Thermo Fisher Scientific, 88518) by wet transfer using NuPAGE transfer buffer (Novex, NP006). Membranes were blocked with 5% bovine serum albumin (BSA) (Thermo Fisher Scientific) in Tris-buffered saline (TBS) at RT for 1h before overnight incubation with primary antibodies at 4^o^C. Membranes were washed 3 × 10 min with TBS-0.1% Tween 20 (TBS-T) before adding anti-rabbit IgG (Cytiva, NA934) and anti-mouse IgG (Cytiva, NA931) HRP-conjugated secondary antibodies diluted 1:10,000. The membranes were washed 3 × 10 min with TBS-T and developed using Bio-Rad ChemiDoc MP. Membranes were incubated for a few seconds with ECL Prime Western blotting detection reagents (Cytiva, RPN2236) to detect proteins. Quantifications were performed using ImageJ/Fiji software.

#### Co-immunoprecipitation

Protein G Sepharose Beads (Cytiva, 17061801) were washed with PBS and then resuspended in IP Buffer [Pierce IP Lysis Buffer (Thermo Fisher Scientific, 89901), phosphatase inhibitor cocktail, protease inhibitor cocktail and 50 nM Na3VO4] diluted in PBS. Cell lysates were pre-cleared by adding beads suspension rotating at 4°C for 1 h. After centrifugation, an aliquot was stored for whole lysate analysis and the rest were incubated with primary antibodies (1:100 dilution) at 4°C for 2 h, rotating. The primary antibodies used for pull-down were all produced in mice and include anti-CSK (Invitrogen Thermo Fisher, MA5-15707), anti-PTPN11 (SHP2) (Origene, TA501914), anti-RASA1 (Thermo Fisher Scientific, MA4-001), anti-VAV2 (Invitrogen Thermo Fisher, MA5-38657), and IgG1 (k isotope) (BD Pharmingen, 555746). Samples were then incubated rotating with beads suspension for 2h at 4°C. Protein complexes were pulled down by centrifugation and washed by resuspending in IP buffer. Proteins were eluted by boiling the samples with NuPAGE LDS Sample Buffer (Invitrogen, NP0007) and NuPAGE Sample Reducing Agent (Invitrogen, NP0009) at 97^o^C, followed by western blotting using rabbit anti-VEGFR2 (2479S) or rabbit anti-PLCγ1 (2822S) antibodies.

#### Proximity ligation assay *in situ*

Proximity Ligation Assay (PLA) was performed according to the instructions provided in the Duolink *In Situ* PLA fluorescence kit and the NaveniFlex Cell kit. In brief, HUVECs were plated at 5 x 10^4^ cells/well in 8-well glass chamber slides. Cells were starved for 2 h, followed by VEGFA stimulation at 100 ng/mL for 5 min at 37°C, or as indicated otherwise. PBS was added to terminate stimulation and cells were fixed with 2% paraformaldehyde (PFA) for 15 min at RT. Cells were permeabilized with TRIS-buffered saline (TBS) (Thermo Fisher Scientific, J60764) 0.1% Triton X-100 for 20 min at room temperature, followed by the blocking step, incubating with the kit’s blocking solution for 1 h at 37°C. Primary antibodies diluted 1:100 in antibody diluent and added for overnight incubation at 4°C include rabbit anti-pSHP2 (Y542) (Cell Signaling, 3751S, 1:250) and mouse anti-PLCγ (E−12) (SantaCruz, sc-7290), rabbit anti–phospho-SFK (Tyr416) (Cell Signaling Technology, 2101s), rabbit anti-phospho-Src (Tyr527) (Cell Signaling Technology, 2105s), mouse anti-Src (Abcam, ab231081), mouse anti-Yes (BD Biosciences, 610376), and mouse anti-3-Nitrotyrosine (Abcam, ab61392). The following day, the slides underwent 2 steps of washing with TBS for 5 min each. PLA probes against rabbit and mouse primary antibodies were added and incubated for 1 h at 37°C. Ligation, rolling circle amplification and detection with fluorescent probes were performed based on the manufacturer’s instructions. The cells were also counterstained with goat-*anti*-VE-cadherin antibody (R&D systems, AF1002; 1:100) and donkey-*anti*-goat-488 (Thermo Fisher Scientific, A-11055; 1:400) as secondary antibody, both diluted in the manufacturer’s antibody diluent.

#### Subcutaneous mouse tumor models

B16F10 melanoma tumor model was established by 1:1 mixing 5 x 10^5^ cancer cells (American Type Culture Collection) with Matrigel (Corning) and then injected subcutaneously into the flank of *Vegfr2*^*Y1173F*^ or *Plcg1*^*iECKO*^ mice at D0, as previously described.^19^ The tumor size was monitored with a caliper every second day from D7 and tumors were collected at D13, to avoid reaching the humane endpoint. Tumor leakage was assessed by intravenously injecting mice with TRITC-70 kDa (Tdb labs) 3 h prior to collection. Mice were then anesthetized using Ketamine/Xylazine and perfused intracardially with DPBS, before tumors were harvested and halved. One-half was immersed in 1% PFA overnight, followed by a 30% sucrose incubation overnight before being embedded in OCT and freeze sectioned (8 μm). The other half was placed in formamide and incubated at 56°C for 48h. Extracted dextran was evaluated using a spectrophotometer, with measurements normalized to weight.

#### The miles assay

Wild-type C57Bl/6 mice (Taconic Biosciences, B6JBOM) were injected intraperitoneally with 4 mg/kg of pyrilamine maleate salt (Sigma, P5514), diluted in 0.9% saline to block histamine release. After 30 min, 100 μL of 1% Evans blue was injected into the tail vein and allowed to circulate in the bloodstream for 10 min. Following anesthesia with 3% isoflurane, DMSO or SHP099 (50 μM) diluted in PBS were administered intradermally into the left and right side of the dorsal skin. Thirty minutes later, 50 μL of either VEGFA164 (100 ng) or PBS were administered to the exact same spots, and 20 min thereafter, the dorsal skin was excised and placed in formamide at 56°C overnight. After 24 h, the absorbance was measured at 620 nm using a spectrophotometer.

#### Immunofluorescence staining

HUVECs and mouse tissue sections were fixed with 1% PFA for 20 min at RT before incubation with blocking buffer: 1X TBS, supplemented with 1% BSA, 0.5% normal donkey serum and 0.1% Triton X- for 1h at RT. Samples were then incubated overnight at 4°C with primary antibodies diluted 1:100 in blocking buffer. The following day, the slides were washed 3 × 10 min with TBS-0.1% Triton X-100 and then incubated for 2h at RT with secondary antibodies conjugated with Alexa Fluor Fluorophores (Thermo Fisher Scientific) diluted 1:400. Slides underwent 3 × 10 min washes with TBS-0.1% Triton X-100, mounted with DAPI Fluoromount-G (SouthernBiotech, 0100-20) and left to air dry overnight under dark conditions. Images were obtained with Leica Confocal Microscope SP8 with objectives HC PL APO CS2 20x/1.30 GLYC HC PL APO CS2 40x/1.30 Oil and HC PL APO CS2 63x/1.30 GLYC. The acquired 1024 x 1024 images were later analyzed by measuring the mean intensity and total area for channels of interest by using Fiji (ImageJ) software.

For immunostainings of human TMAs, slides were initially deparaffinized by 2 x 5-min incubations in fresh xylene, followed by rehydration in decreasing concentrations of ethanol (100%, 95%, 70%) for 2 × 5 min each and 5-min incubation with ddH2O. Heat-induced epitope retrieval (HIER) was conducted by incubating the slides with target retrieval solution, citrate pH6.1 (DAKO, S1699) in a steamer cycle of pressure boiling for 20 min, subsequent by cooling down for 2 h. Blocking was performed for 1 h at room temperature using serum-free protein blocking buffer (DAKO, X0909). Slides were then incubated overnight at 4°C with primary antibodies diluted 1:100 in DAKO blocking buffer. After washing the slides three times 10 min each with 1X TBS +0.05% Tween 20, TMA samples were incubated for 1 h at room temperature with secondary antibodies diluted 1:300, prior to mounting and air-drying under dark conditions. A relative score for vascular SHP2 expression into low and high levels was performed by two investigators, independently.

#### Multiplex immunofluorescence and multispectral imaging

TMA slides were used for multiplex immunofluorescence and multispectral imaging according to previously described protocol by Nwadozi et al.^36^ Multiplex staining was conducted in accordance with the protocol outlined in the Akoya Biosciences Opal Multiplex IHC Assay Development Guide. The sequential procedures were carried out according to antibody and fluorophore specifications and they included blocking with BLOXALL Endogenous Blocking Solution (Vector Labs, SP-6000-100), application of the primary antibody diluted in blocking buffer, HRP polymer detection utilizing ImmPRESS-VR horse anti-mouse/rabbit IgG (Vector Lab, MP-6402-15 and MP-7801-15), signal amplification using TSA-Opal fluorophores diluted in amplification buffer, and subsequent washing with 1xTBS +0.05% Tween 20 (Sigma Aldrich, P1379). For each sequence, the primary-secondary-HRP complex was ejected by performing HIER. After the last round, samples were counterstained with Hoechst 33342, mounted and left to dry overnight. Multispectral slide scans were conducted using the Vectra Polaris Automated Quantitative Pathology Imaging System (Akoya Biosciences) at 20× magnification. A relative score for vascular PLCγ- or SHP2 protein expression levels, as well as extravascular FpA immunoreactivity, into low, medium and high was primarily performed by two independent investigators. A consensus score was established in cases of a different score.

### Quantification and statistical analysis

Statistical analysis was performed with GraphPad Prism 10 software by comparing the means of at least 3 independent biological replicates of each experiment. Statistical significance was calculated using unpaired 2-tailed Student’s *t* test, 1-way or two-way ANOVA with Tukey’s multiple comparisons test. Data are expressed as mean ± SD and a *p-value* < 0.05 was considered statistically significant, indicated by ∗*p* < 0.05, ∗∗*p* < 0.01, ∗∗∗*p* < 0.001. χ2 test was used to determine associations of PLCγ or SHP2 vascular protein expression and FpA staining (vascular leakage) in scored human TMA samples. A *p-value* < 0.05 was considered statistically significant. For animal experiments, no statistical methods were used to predetermine the sample size. The investigators were blinded to allocation during experiment and outcome assessment.
