# Supplementary material for: SHP2 regulates VEGFR2 Y1175/PLCγ signaling to impair tumor endothelial barrier stability
Source: iScience. 2026 Jan 27;29(2):114784. doi: 10.1016/j.isci.2026.114784 (PMC12915272; doi:10.1016/j.isci.2026.114784)
Supplement: Document S1. Figures S1–S6 [file mmc1.pdf]

iScience, Volume *29*

Supplemental information

SHP2 regulates VEGFR2 Y1175/PLCγ signaling to impair tumor endothelial barrier stability

Polina Kremmyda, Sara Owad, Sagnik Pal, Elvira Wildheim, Catarina Chanoca, Cecilia Lindskog, and Elin Sjöberg

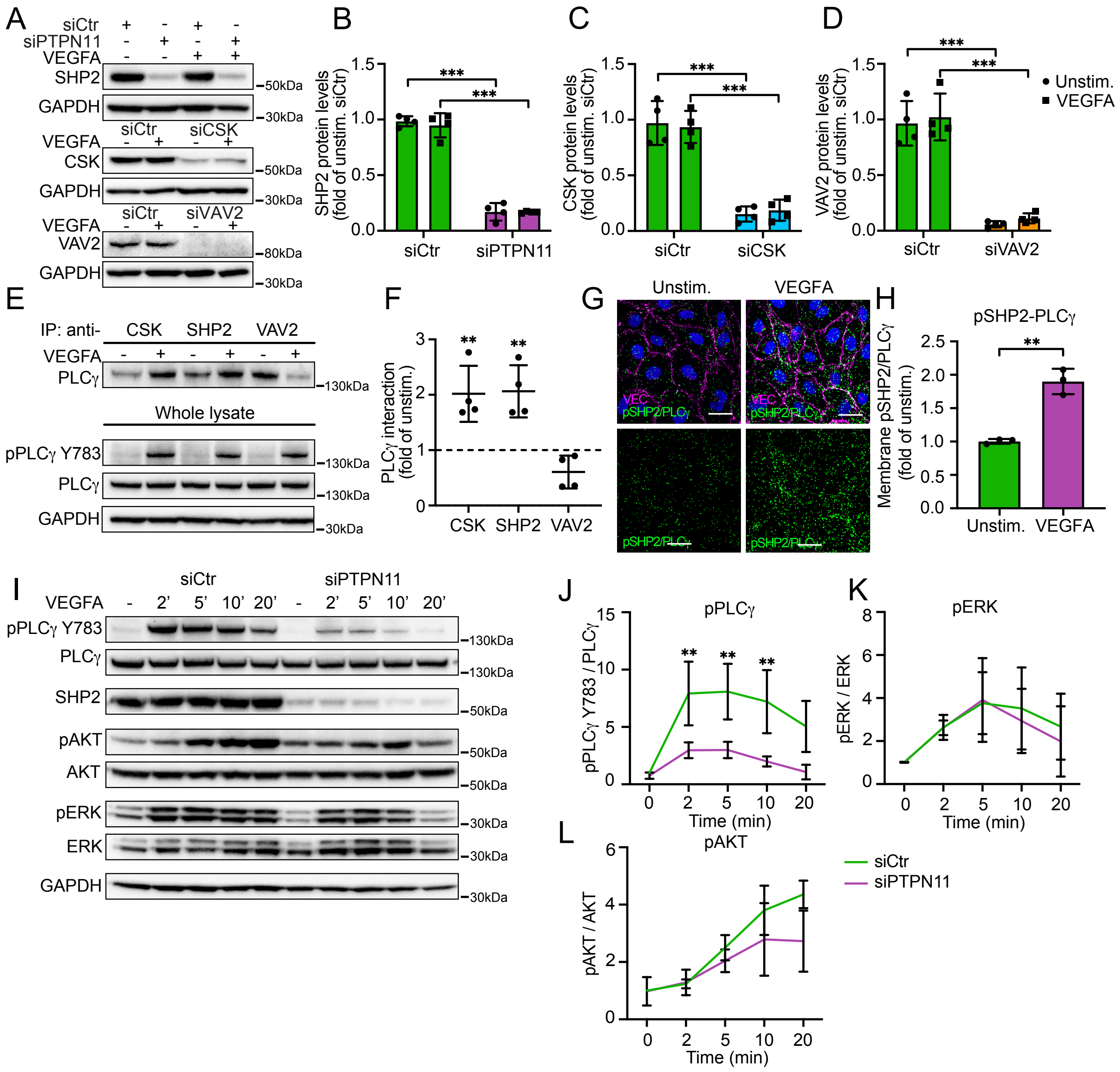

Figure S1: SHP2 is identified as a novel PLCγ-dependent VEGFR2 pY1175 interaction partner in endothelial cells, related to Figure 1

(A) Western blot validating the efficiency of *siPTPN11*, *siCSK* and *siVAV2* downregulation in unstimulated (-) or 100 ng/mL VEGFA-stimulated (+) HUVECs, using antibodies against SHP2, CSK or VAV2 respectively.

(B-D) Quantifications of protein levels from A, for SHP2 (B), CSK (C) and VAV2 (D), shown as fold change of untreated siCtr; $n = 4$ independent experiments.

(E) Representative Western blot showing PLCγ immunoprecipitated with antibodies against CSK, SHP2 or VAV2 in unstimulated (-) or 100 ng/mL VEGFA-stimulated (+) HUVECs. Corresponding whole-cell lysates were assessed by blotting for pPLCγ Y783, total PLCγ, and GAPDH as a loading control.

(F) Quantification of E, for PLCγ interactions with CSK, SHP2 and VAV2, shown as fold change to unstimulated control; $n = 4$ independent experiments.

(G and H) Representative proximity ligation assay (PLA) (G) and quantification (H) of pSHP2/PLCγ complexes in unstimulated or VEGFA-stimulated (100 ng/mL, 5 min) HUVECs, using antibodies against pSHP2 Y542 and PLCγ. Junctions are stained for VE-cadherin (VEC; magenta) and nuclei for DAPI (blue). Scale bar: 30 µm; $n = 3$ independent experiments.

(I) Representative Western blot of unstimulated (-) or 100 ng/mL VEGFA-stimulated HUVECs for 2, 5, 10 and 20 minutes, pretreated with *siCtr* or *siPTPN11*, using antibodies against phospho- and total proteins of PLCγ, AKT and ERK.

(J-L) Quantification of Western blots from I, for activation of PLCγ (J), ERK (K) and AKT (L), shown as fold change of unstimulated control; $n = 4$ independent experiments.

One-way ANOVA. Data represent mean ± SD. $^{**}p < 0.01$, $^{***}p < 0.001$.

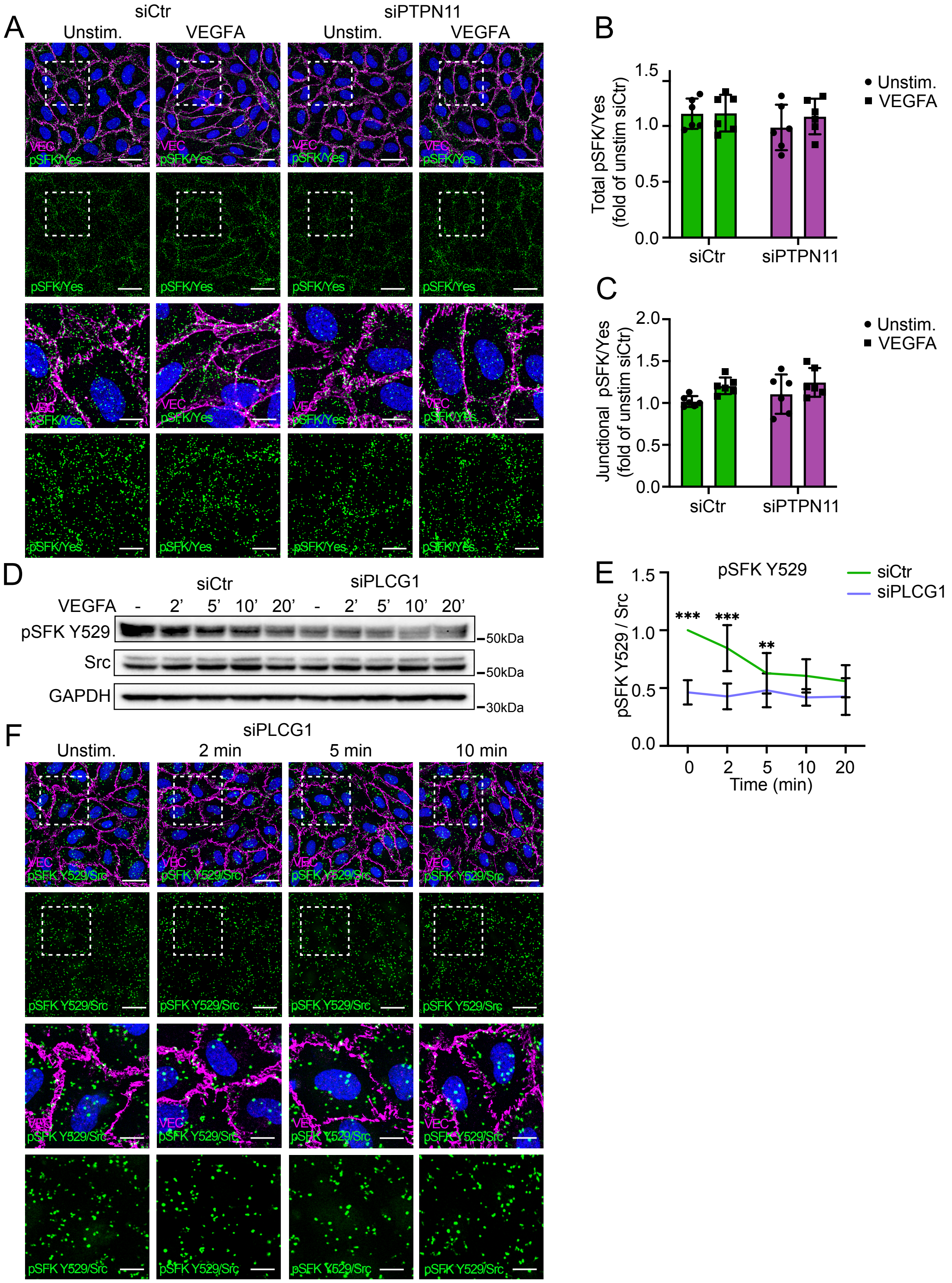

Figure S2: Endothelial PLCγ/SHP2-signaling mediate activation of Src by regulation of both the activating and inhibitory tyrosine phosphorylation sites, related to Figure 2

(A) Proximity ligation assay (PLA) using antibodies against Yes and pSFK Y418 to detect Yes phosphorylation at Y418 in unstimulated or VEGFA-stimulated (100 ng/mL, 5 min) HUVECs, pretreated with *siCtr* or *siPTPN11*. Endothelial junctions are stained for VE-cadherin (VEC; magenta) and nuclei for DAPI (blue). Scale bar: 30 µm. Boxed regions in the upper panels are shown at higher magnification in panels below. Scale bar: 10 µm.

(B and C) MFI quantifications from A, for total (B) and junctional (C) PLA signals, representing Y418 phosphorylation of Yes; n = 6 independent experiments, ≥ 3 fields of view per experiment.

(D) Representative Western blot showing pSFK Y529 signaling in untimulated (-) or 100 ng/mLVEGFA-stimulated HUVECs for 2, 5, 10 and 20 minutes, pretreated with *siCtr* or *siPLCG1*.

(E) Quantification of Western blots from D; n = 5 independent experiments.

(F) PLA using antibodies against Src and pSFK Y529, visualizing phosphorylation of Src at the inhibitory phosphosite in untimulated or 100 ng/mL VEGFA-stimulated HUVECs for 2, 5 and 10 minutes, pre-treated with *siPLCG1*. Endothelial junctions are stained for VEC (magenta) and nuclei for DAPI (blue). Scale bar: 30 µm. Boxed regions in the upper panels are shown at higher magnification in panels below. Scale bar: 10 µm.

One-way ANOVA. Data represent mean ± SD. $^{**}p < 0.01$, $^{***}p < 0.001$.

.

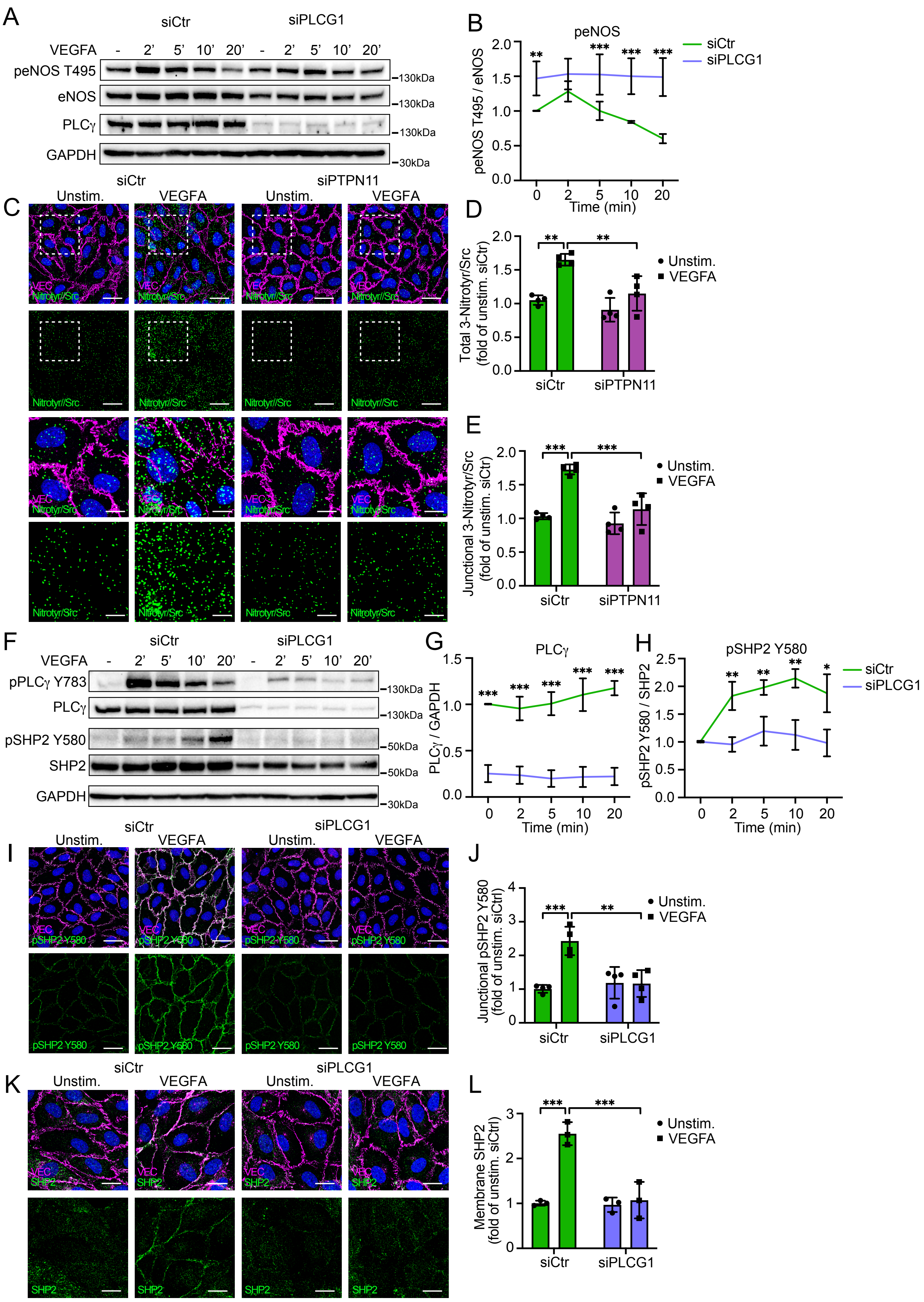
A
siCtr
siPLCG1
VEGFA - 2' 5' 10' 20' - 2' 5' 10' 20'
peNOS T495
eNOS
PLCγ
GAPDH
130kDa
130kDa
130kDa
30kDa
B
peNOS
siCtr
siPLCG1
peNOS T495 / eNOS
Time (min)
C
siCtr
siPTPN11
Unstim.
VEGFA
VEC
Nitrotyr//Src
Nitrotyr/Src
D
Total 3-Nitrotyr/Src (fold of unstim. siCtr)
Unstim.
VEGFA
siCtr
siPTPN11
E
Junctional 3-Nitrotyr/Src (fold of unstim. siCtr)
Unstim.
VEGFA
siCtr
siPTPN11
F
siCtr
siPLCG1
VEGFA - 2' 5' 10' 20' - 2' 5' 10' 20'
pPLCγ Y783
PLCγ
pSHP2 Y580
SHP2
GAPDH
130kDa
130kDa
50kDa
50kDa
30kDa
G
PLCγ
PLCγ / GAPDH
Time (min)
H
pSHP2 Y580
siCtr
siPLCG1
pSHP2 Y580 / SHP2
Time (min)
I
siCtr
siPLCG1
Unstim.
VEGFA
VEC
pSHP2 Y580
J
Junctional pSHP2 Y580 (fold of unstim. siCtrl)
Unstim.
VEGFA
siCtr
siPLCG1
K
siCtr
siPLCG1
Unstim.
VEGFA
VEC
SHP2
L
Membrane SHP2 (fold of unstim. siCtrl)
Unstim.
VEGFA
siCtr
siPLCG1

Figure S3: PLCγ/SHP2 interplay leads to eNOS activation followed by Src nitration, related to Figure 3

(A) Representative Western blot showing eNOS T495 signaling in unstimulated (-) or 100ng/mL stimulated HUVECs for 2, 5, 10 and 20 minutes, pre-treated with *siCtr* or *siPLCG1*.
(B) Quantification of Western blots from A; $n = 6$ independent experiments.
(C) PLA for NitroTyr and Src to detect nitration of Src in HUVECs stimulated with VEGFA (100 ng/mL, 5 min) or left unstimulated, and pretreated with *siCtr* or *siPTPN11*. Endothelial junctions are stained for VE-cadherin (VEC; magenta) and nuclei for DAPI (blue). Scale bar: 30 µm. Boxed regions in the upper panels are displayed in higher magnification in panels below. Scale bar: 10 µm.
(D and E) Quantification of PLAs from C, for total (D) and junctional (E) MFI PLA signals representing 3-Nitration of Src; $n = 4$ independent experiments, ≥ 3 fields of view per experiment.
(F) Representative Western blot with antibodies against pPLCγ Y783 and pSHP2 Y580 in unstimulated (-) or 100ng/mL stimulated HUVECs for 2, 5, 10 and 20 minutes, pretreated with *siCtr* or *siPLCG1*.
(G and H) Quantifications of Western blots from E, for PLCγ (G) and pSHP2 Y580 (H); $n > 3$ independent experiments.
(I and J) Representative immunostainings (I) and quantification (J) of unstimulated or VEGFA-stimulated HUVECs (100 ng/mL, 5 min), pretreated with *siCtr* or *siPLCG1*, stained for pSHP2 (Y580) (green) and VEC (magenta). Scale bar: 30 µm; $n = 4$ independent experiments, ≥ 3 fields of view/experiment.
(K and L) Representative immunostainings (K) and quantification (L) of unstimulated or VEGFA-stimulated HUVECs (100 ng/mL, 5 min), pretreated with *siCtr* or *siPLCG1*, stained for SHP2 (green) and VEC (magenta). Scale bar: 20 µm; $n = 4$ independent experiments, ≥ 3 fields of view/experiment.
One-way ANOVA. Data represent mean ± SD. *$p < 0.05$, **$p < 0.01$, ***$p < 0.001$.

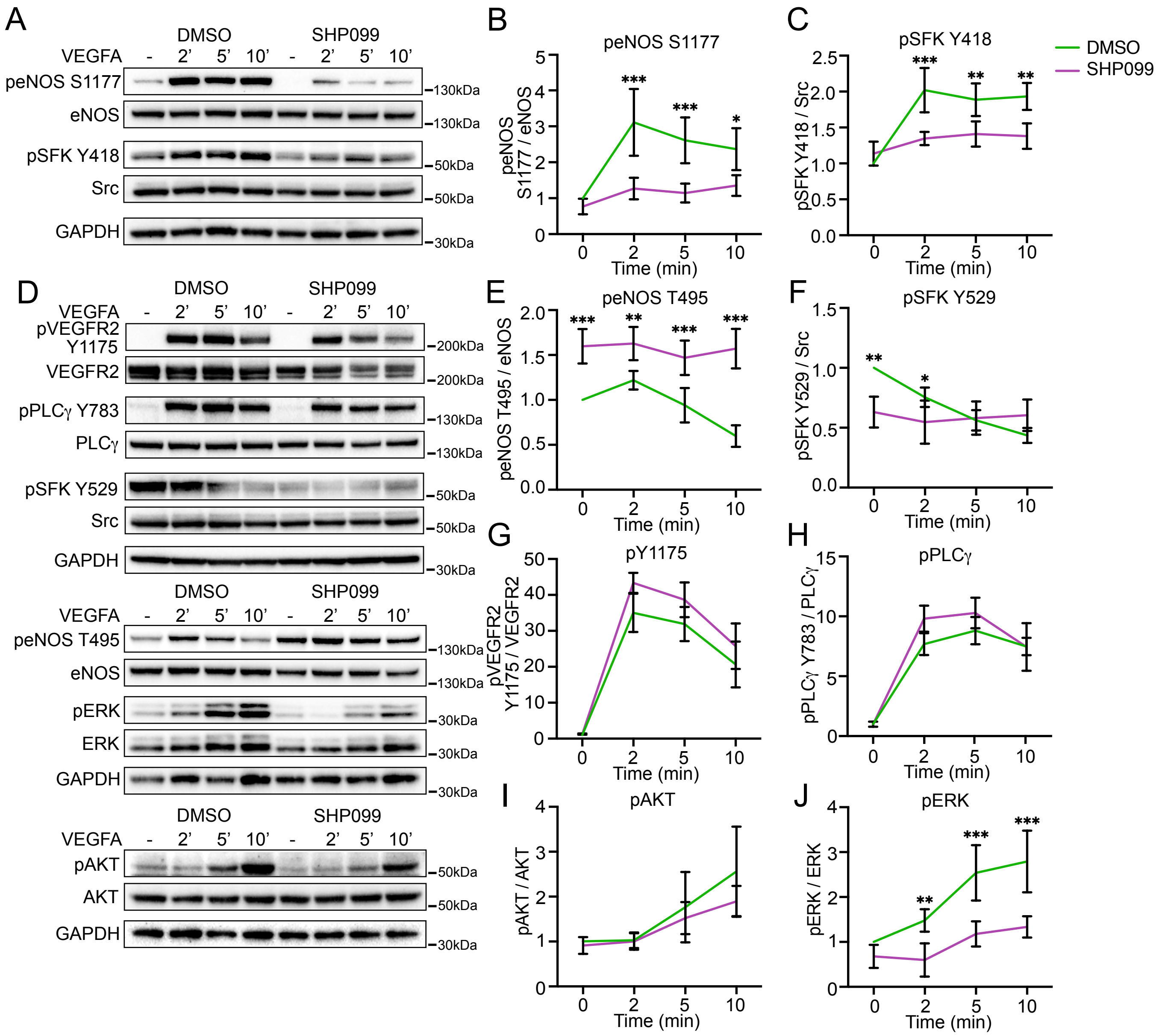

Figure S4: Pharmacological inhibition of SHP2 reduces VEGFA-stimulated Src and eNOS activation, related to Figure 4

(A) Representative Western blot of unstimulated (-) or 100 ng/mL VEGFA-stimulated HUVECs for 2, 5 and 10 min, pre-treated with DMSO (control) or the allosteric SHP2 inhibitor SHP099 (30 μM) for 4 hours.

(B and C) Quantifications of Western blots from A, for peNOS S1177 (B) and pSFK Y418 (C); *n* = 5–6 independent experiments.

(D) Representative Western blot showing VEGFR2 downstream signaling in unstimulated (-) or 100 ng/mL VEGFA stimulated HUVECs, pretreated with DMSO (control) or the allosteric SHP2 inhibitor SHP099 (30 μM) for 4 hours.

(E-J) Quantifications of Western blots from D, for peNOS T495 (E), pSFK Y529 (F), pVEGFR2 Y1175 (G), pPLCγ Y783 (H), pAKT (I) and pERK (J); *n* = 3–6 independent experiments.

One-way ANOVA. Data represent mean ± SD. *$p < 0.05$, **$p < 0.01$, ***$p < 0.001$.

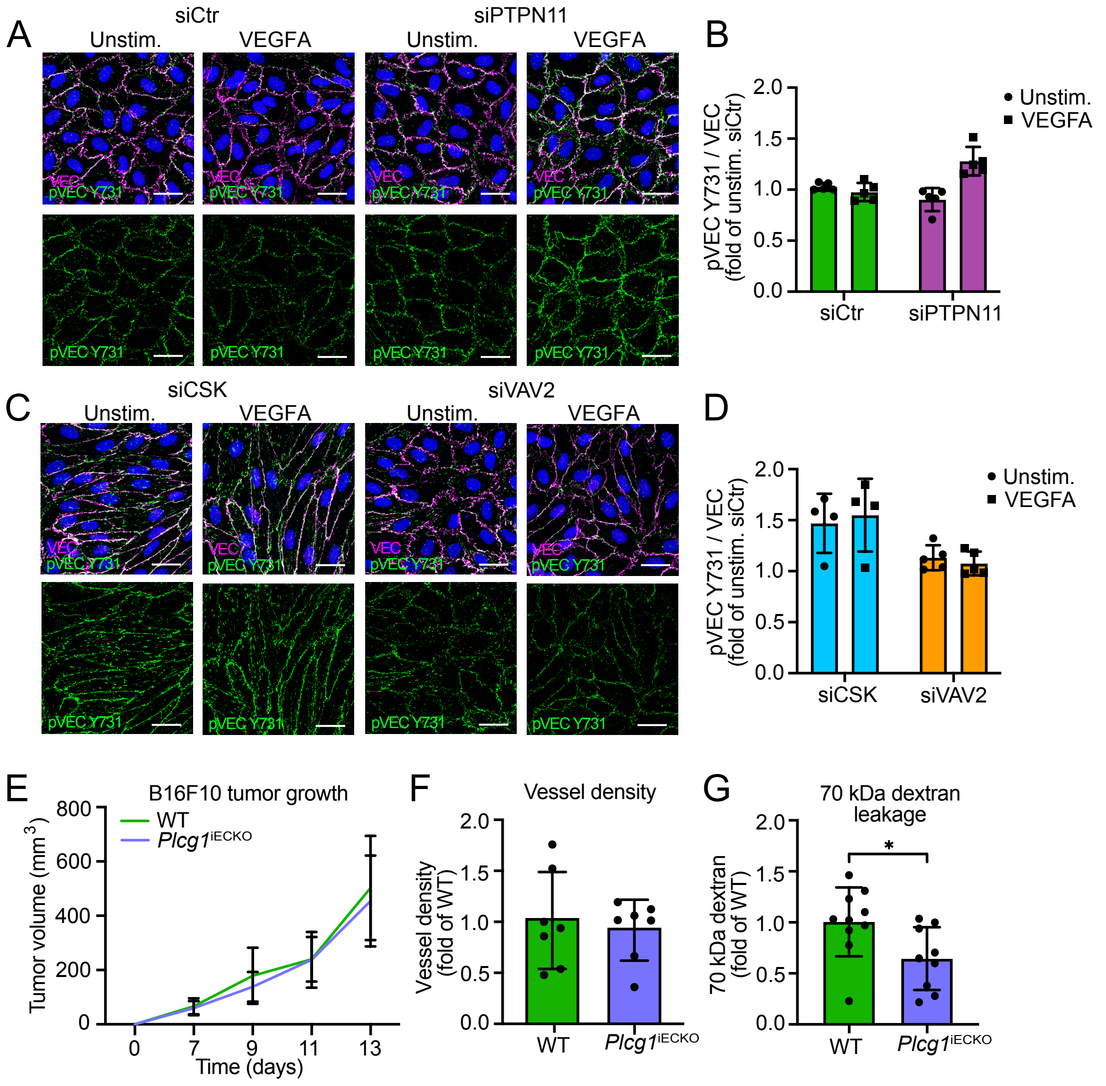

Figure S5: VEGFR2 Y1173 heterozygosity is accompanied by decreased tumor endothelial PLCγ/SHP2 signaling and tumor vascular leakage, related to Figure 5

(A) Representative images of immunostainings for VE-cadherin (VEC; magenta), pVEC Y731 (green) and DAPI (blue) of unstimulated or VEGFA-stimulated (100 ng/mL, 5 min) HUVECs, pretreated with *siCtr* or *siPTPN11*. Scale bar: 30 μm.
(B) Quantification of MFI from A, shown as fold of unstimulated control; *n* = 5 independent experiments, ≥ 3 fields of view/experiment.
(C) Immunostaining of unstimulated or VEGFA-stimulated HUVECs (100 ng/mL, 5 min), pretreated with *siCSK* or *siVAV2*, using antibodies against VE-cadherin (VEC; magenta), pVEC Y731 (green) and DAPI (blue). Scale bar: 30 μm.
(D) Quantification of MFI from C, shown as fold of unstimulated siCtr; *n* = 4 (*siCSK*) and *n* = 5 (*siVAV2*) independent experiments, ≥ 3 fields of view/experiment.
(E) Tumor growth of B16F10 melanoma in wild-type (WT) and *Plcg1*^{iECKO} mice.
One-way ANOVA. Data represent mean ± SD.
(F) Quantification of vessel density (MFI) from Figure 5G; *n* = 7 mice per genotype. ≥ 3 fields of view/experiment.
(G) Fluorescent intensity of 70 kDa TRITC-dextran extracted from B16F10 tumors; *n* = 10 (WT) and 9 (*Plcg1*^{iECKO}) mice. Unpaired 2-tailed students' t-test. Data represent mean ± SD. *$p < 0.05$.

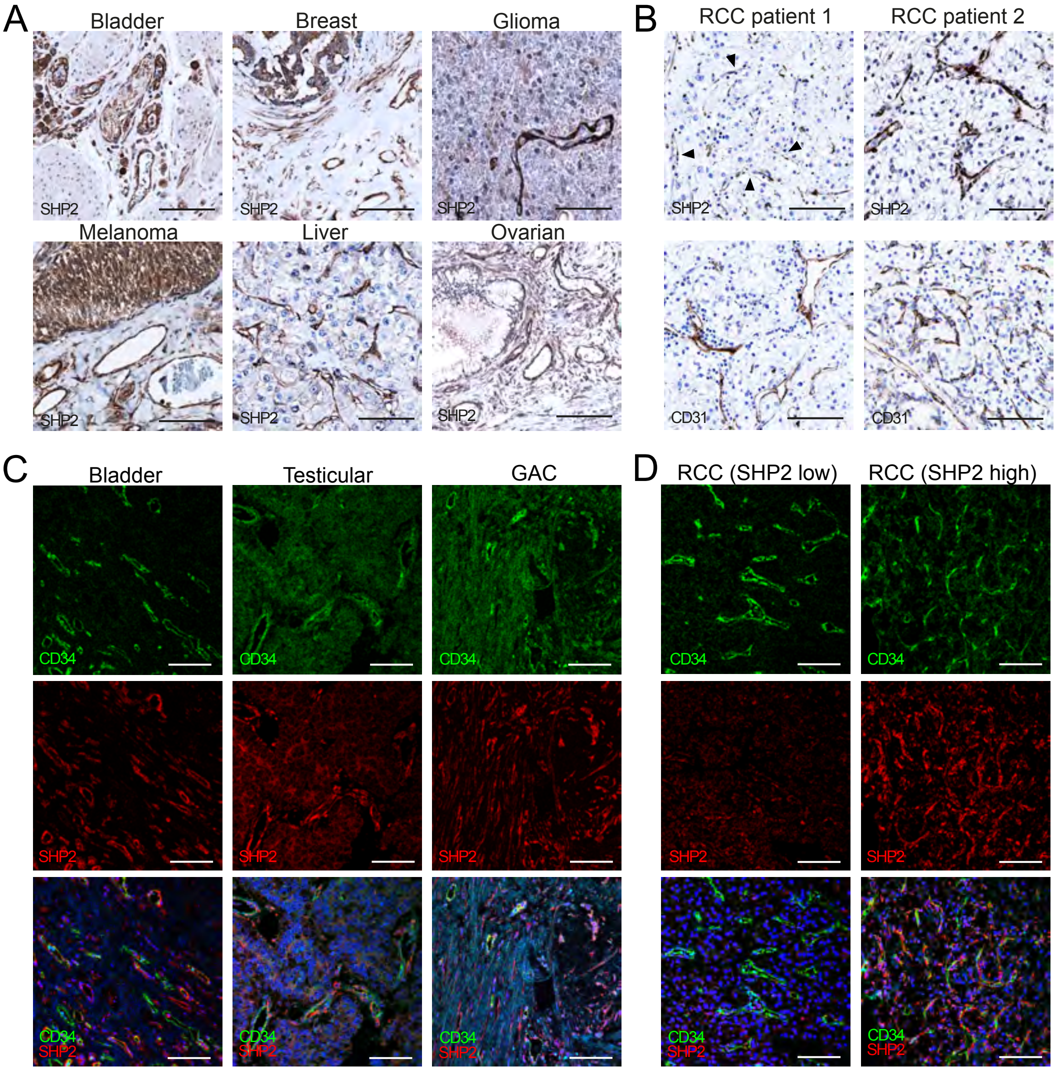

Figure S6: SHP2 is a clinically relevant biomarker for tumor vascular leakage, related to Figure 6

(A and B) Immunohistochemistry (IHC) stainings by the Human Protein Atlas (HPA) consortium of SHP2 expression in human tumor tissue samples derived from bladder, breast, glioma, melanoma, liver, ovarian cancer (A) and renal cell carcinoma (RCC) (B). Scale bar: 100 µm.

(C and D) Immunofluorescent (IF) stainings for CD34 (green), SHP2 (red) in human tumor samples from bladder, testicular, gastrointestinal adenocarcinoma (GAC) (C) and RCC (D) with low and high endothelial SHP2 expression. Nuclei are stained for DAPI (blue). Scale bar: 100 µm.
